# Dissection of the molecular bases of genotype x environment interactions: a study of phenotypic plasticity of *Saccharomyces cerevisiae* in grape juices

**DOI:** 10.1186/s12864-018-5145-4

**Published:** 2018-11-09

**Authors:** Emilien Peltier, Vikas Sharma, Maria Martí Raga, Miguel Roncoroni, Margaux Bernard, Vladimir Jiranek, Yves Gibon, Philippe Marullo

**Affiliations:** 1 Univ. Bordeaux, ISVV, Unité de recherche OEnologie EA 4577, USC 1366 INRA, Bordeaux INP, Villenave d’Ornon, France; 2 Biolaffort, Bordeaux, France; 3 Departament de Bioquímica i Biotecnologia, Facultat d’Enologia de Tarragona, Tarragona, Spain; 4 Wine Science Programme, University of Auckland, Private Bag, Auckland, 92019 New Zealand; 5 Department of Wine and Food Science, University of Adelaide, Urrbrae, South Australia 5064 Australia; 6 INRA, University of Bordeaux, UMR 1332 Fruit Biology and Pathology, F-33883 Villenave d’Ornon, France

**Keywords:** Yeast, QTL mapping, Fermentation, Gene*–*environment interaction, Enology

## Abstract

**Background:**

The ability of a genotype to produce different phenotypes according to its surrounding environment is known as phenotypic plasticity. Within different individuals of the same species, phenotypic plasticity can vary greatly. This contrasting response is caused by gene-by-environment interactions (GxE). Understanding GxE interactions is particularly important in agronomy, since selected breeds and varieties may have divergent phenotypes according to their growing environment. Industrial microbes such as *Saccharomyces cerevisiae* are also faced with a large range of fermentation conditions that affect their technological properties. Finding the molecular determinism of such variations is a critical task for better understanding the genetic bases of phenotypic plasticity and can also be helpful in order to improve breeding methods.

**Results:**

In this study we implemented a QTL mapping program using two independent cross (~ 100 progeny) in order to investigate the molecular basis of yeast phenotypic response in a wine fermentation context. Thanks to whole genome sequencing approaches, both crosses were genotyped, providing saturated genetic maps of thousands of markers. Linkage analyses allowed the detection of 78 QTLs including 21 with significant interaction with the environmental conditions. Molecular dissection of a major QTL demonstrated that the sulfite pump Ssu1p has a pleiotropic effect and impacts the phenotypic plasticity of several traits.

**Conclusions:**

The detection of QTLs and their interactions with environment emphasizes the complexity of yeast industrial traits. The validation of the interaction of *SSU1* allelic variants with the nature of the fermented juice increases knowledge about the impact of the sulfite pump during fermentation. All together these results pave the way for exploiting and deciphering the genetic determinism of phenotypic plasticity.

**Electronic supplementary material:**

The online version of this article (10.1186/s12864-018-5145-4) contains supplementary material, which is available to authorized users.

## Background

Phenotypic plasticity, which is the ability of a genotype to produce distinct phenotypes in different environmental conditions, has been widely reviewed [1–3]. It encompasses various aspects of the organism’s life including ontogeny [4], lifespan [5, 6], response to biotic [7, 8] or abiotic [9, 10] factors, pathogen or disease susceptibility [11–13], and animal behavior [12, 14]. As a universal mechanism, phenotypic plasticity has been reported in humans [12], animals [6, 15, 16], plants [4], and fungi [17, 18]. The term of phenotypic plasticity can be used at different integrative levels. At the population level the phenotypic plasticity is the overall phenotypic response of a species to different environments. The genetic basis of those responses is mainly explained by transcriptional [19, 20], post-transcriptional [21], and/or epigenetic [12, 17] regulations. Phenotypic plasticity can also be considered at the individual level within a species [1, 3]. In this case the phenotypic plasticity is measured for specific genotypes. The phenotypic response pattern observed is termed the reaction norm. Among individuals of the same species, non-parallel reaction norms are often observed in animals [22, 23], plants [24, 25] and fungi [26]. These different patterns of response are due to the genotype-by-environment interaction (GxE) determined by allelic variations having different effects according to external conditions. The genetic bases of GxE interactions can be investigated at a genomic level by QTL mapping programs or genome-wide association studies carried out in various environmental conditions. These two strategies have commonly been used for animals [27–29], plants [30–32] and QTL mapping for fungi [33–36]. Although many QTLs interacting with environment are often detected, the identification of the genetic basis of GxE at a gene level is far from being trivial. Therefore only few molecular evidences has been reported in plants [37, 38] and yeast [39, 40].

In agronomy, the concept of phenotypic plasticity has been integrated a long time ago to ensure the stability (robustness) of domesticated plant varieties and animal races across diverse uncontrollable macro environments [22, 41–43]. Apart from species of agronomic interest, fungi and in particular yeast are eukaryotic organisms with an important economic impact. The bakers’ yeast *Saccharomyces cerevisiae*, is by far the major industrial microorganism since it is involved in production of numerous fermented foods including bread, wine and beer [44]. These processes can be better controlled, by using industrial starters that have been subjected to genetic selection using breeding programs [45]. Depending on the composition of the fermentation matrix, yeasts are faced with various stresses and conditions inducing contrasting technological response. In winemaking, the initial composition of the grape must strongly impacts the alcoholic fermentation. Indeed, grape cultivars, enological practices, *terroir* and climate modulate biotic (populations size of various species including yeasts, molds, bacteria) and abiotic (sugar, nitrogen, lipid, vitamin concentration, oxygen, temperature, turbidity) factors and strongly shape the phenotypic variability of wine yeasts [46–55]. Understanding how and why industrial starters have non-parallel norms of reaction is a critical challenge for industrial yeast selection. Understanding phenotypic robustness (also named canalization or homeostasis) is of great importance for selecting more robust individuals able to ensure successful fermentation in a wide range of conditions. Recently the dissection of fermentation kinetics QTLs demonstrated that two genes involved in pH homeostasis showed GxE interaction according to wine pH [40]. In the present work, we applied a QTL mapping program aiming to identify QTLs interacting with environmental conditions. Eleven quantitative traits related to alcoholic fermentation were measured for two distinct populations of ~ 100 progeny each in three distinct conditions simulating diverse winemaking practices. The use of two independent backgrounds allowed estimation of the impact of parental divergence on QTL identification. High-density genetic maps generated by genome sequencing enabled QTLs to be detected at the gene level. Although most of the QTLs were robust to environmental changes, some striking GxE interactions were identified. One of these was explained at the molecular level revealing that the sulfite pump Ssu1p has a strong pleiotropic and plasticity role in wine fermentation.

## Results

### Experimental design to capture GxE

**Fig. 1:**
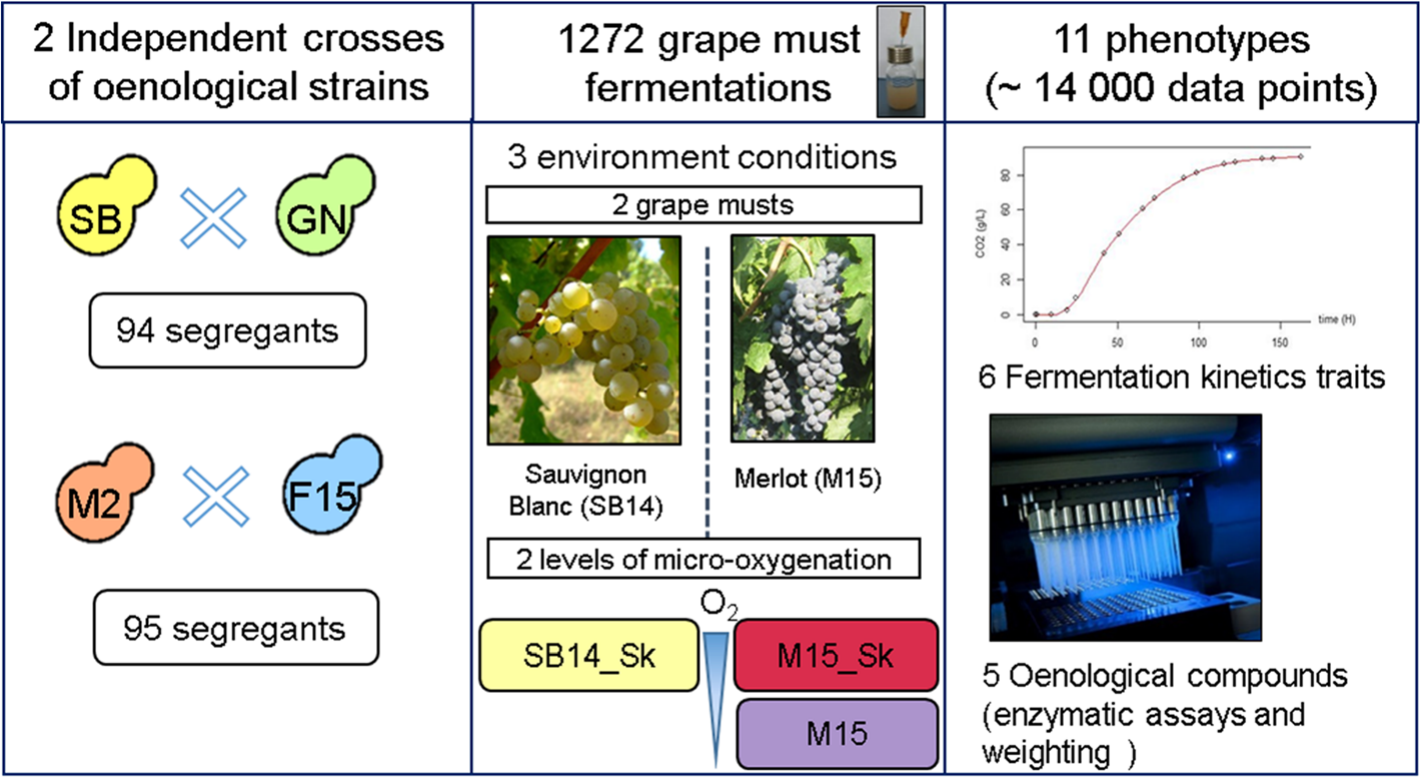
Experimental design. Four diploid homozygous strains (SB, GN, F15, M2) were used to generate two independent hybrids (SBxGN and M2xF15) and their 94 and 95 segregants, respectively. The whole genome sequence of each parental strain and each progeny was obtained by high throughput sequencing and analyzed to get saturated genetic maps (3433 and 8378 markers, respectively). The phenotypic characterization of the strains was carried out in three conditions (SB14_Sk, M15_SK and M15) in small volume vessels. Fermentation time course was monitored and six traits were computed: lag phase (*lp*), time required to produce 35, 50 and 80 g.L^− 1^ of CO_2_ (*t35 g*, *t50 g* and *t80 g,* respectively) and glucose consumption rate in the first and the second part of the fermentation (*V15_50* and *V50_80*, respectively). The concentrations of five end-point metabolites were also assayed by enzymatic methods (*acetic acid*, *glycerol*, *pyruvate* and *SO*_*2*_) or by weighting (*CO*_*2*_*max*). Grape pictures were reprinted from CC-BY licences, the copyright holders being Lebowskyclone (Merlot) and User:Vl (Sauvignon Blanc). Machinery picture is own by the authors

The purpose of this study was to find out at a large scale QTLs interacting with the environment by using the model yeast *S. cerevisiae.* As QTLs can be readily used in yeast breeding for strain improvement, this work was carried out in an enological context by measuring the phenotypic plasticity of wine related strains in three contrasting conditions met in winemaking. Two particular effects were investigated: (i) the phenotypic plasticity of yeast fermenting red or white grape musts (GM) was estimated by comparing M15_Sk (red) vs SB14_Sk (white) conditions, reflecting common types of grape juice fermented around the world. (ii) the phenotypic response to micro-oxygenation (μ-Ox) in accordance with enological practices [56] was estimated by comparing M15_Sk (shaken) vs M15 (unshaken). In order to have the broadest landscape of phenotypic plasticity, we performed QTLs mapping in two distinct genetic backgrounds derived from commercial starters widely used in industry (SB, GN, M2 and F15). The full experimental design is summarized in Fig. 1. With this dataset, we addressed a main question: we investigated if yeast strains display different norms of reaction according to the grape juice and the fermentation condition used and if we can identify the causative genetic determinants. By performing the experiment with two independent crosses, we also had the opportunity to evaluate the impact of the genetic and/or phenotypic characteristics of parental strains on the architecture of quantitative trait determinism.

### Parental pairs have divergent profile at the phenotypic and genetic level

**Fig. 2:**
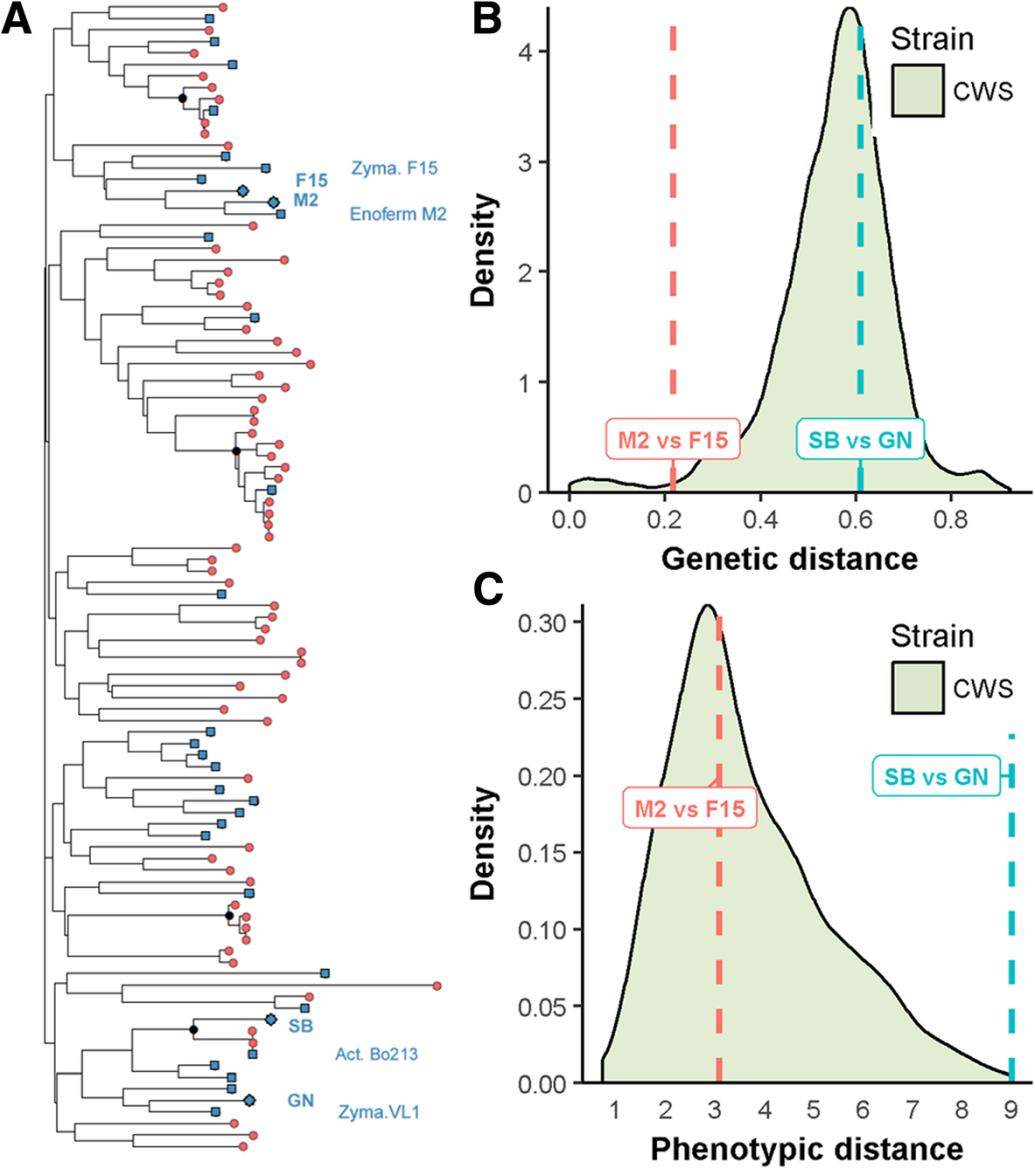
Genetic distance between parental strains. Panel **a** Genetic relationships between 96 commercial wine yeast strains and the four parental monosporic clones GN, M2, F15 and SB used in this work (diamonds). The dendrogram tree was built using Bruvo’s distance and Neighbor-Joining’s clustering strains from the inheritance of 15 polymorphic microsatellites described by Legras et al. [55]. Black dots represents nodes encompassing group of strains highly similar (bootstrap values > 85). Among the strains genotyped, 31 (CWS) have been previously phenotyped and covered all the branches of the tree (blue squares). Panel **b** The distribution of Bruvo’s distance for all genotyped strains. The relative distances between SB vs GN and M2 vs F15 are 0.61 and 0.22, respectively. Panel **c** The distribution of the phenotypic distance computed for 35 strains (31 CWS and the four parental strains). The data used eight traits in five grape musts and were obtained from [56]. Dashed lines shows the relative distance between parental pairs SB vs GN and M2 vs F15

The parental strains were phenotypically and genetically characterized with other Commercial Wine Starters (CWS). The goal is to position these strains among their counterparts. In order to estimate the genetic relationships between the four parental strains, 15 polymorphic microsatellite markers were used [57]. The codominant allele set of each parental strain was compared to those of 96 CWS encompassing the overall diversity of the *S. cerevisiae* wine group (Additional file 1: Table S1). This dataset was used to build a tree using Bruvo’s distance (Fig. 2a). The pairwise Bruvo’s genetic distance between all the strains ranged from 0 (identical strains) to 0.924, with an average of 0.552. According to previous studies, the wine strain population is poorly structured. Four groups of strains (nodes with bootstrap values higher than 85%) were found. One of these groups corresponds to the Champagne-like strains, which have specific genetic features [57, 58]. A second encompasses the strain Actiflore Bo213 and the parental strain SB. As expected, the parental monosporic clones (SB, GN, M2, F15) are closely related to their respective commercial ancestors (Actiflore BO213, Zymaflore VL1, Enoferm M2 and Zymaflore F15, respectively). Considering the overall genetic diversity of the *S. cerevisiae* species, the wine yeast cluster constitutes quite a homogenous group [57, 58]. However, within this group the genetic relationships between parental pairs are different. The strains GN and SB are more divergent than the strains M2 and F15 (Bruvo’s genetic distance = 0.611 and 0.216, respectively; Fig. 2b). Similar results were obtained by calculating the % identity of a subset of 5281 polymorphic SNPs extracted from parental genomes (Sharma, personal communication). The relative phenotypic distance between parental strains was estimated using the phenotypic values of eight fermentation traits measured in five grape musts for 35 strains previously obtained [56]. In order to represent the genetic diversity of commercial starters, the sampled strains belong to the different branches of the tree. The pairwise Euclidian’s phenotypic distance between all the strains ranges from 0.73 (similar strains) to 9, with an average of 3.7. The strains SB and GN are phenotypically very different (9) while F15 and M2 have more similar phenotypes (3). All together, these results demonstrate that the hybrids used for QTL mapping (M2xF15 and SBxGN) were obtained from independent strains showing either low or medium genetic distances and medium or high phenotypic distances.

### Meiotic recombination emphasizes phenotypic variability in either tested cross

**Fig. 3:**
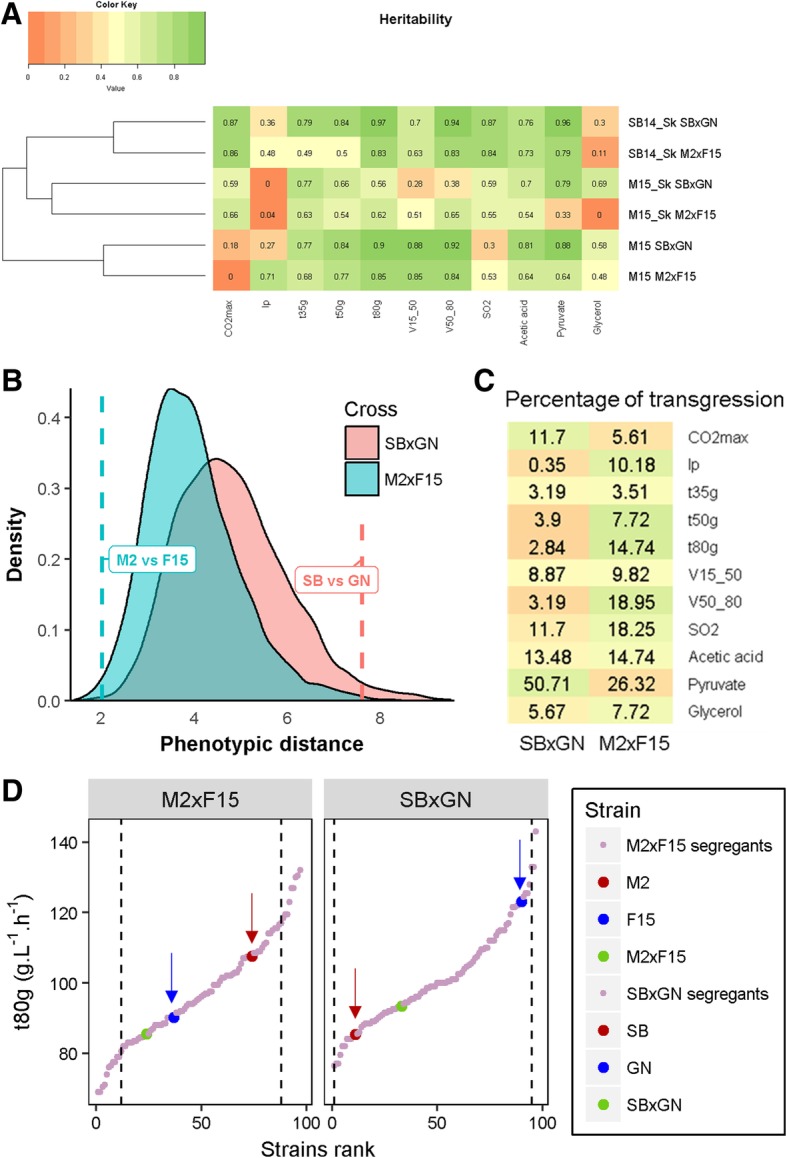
Phenotypic distribution patterns in the two progeny populations. Panel **a** Heritability calculated for each trait according to cross and condition. Panel **b** Dashed lines show the phenotypic distance between parental strains. Shaded areas show the distribution of the phenotypic distance within progenies. Panel **c** Average percentage of transgression per trait in the three conditions according to the cross. Color key is scaled by trait, dark green means high transgression level and dark red low transgression level. Panel **d** The distributions of *V50_80* (M15_Sk condition) illustrate the transgression discrepancy observed between the two crosses. Each dot represents a strain value ordered according to the rank. Parental phenotypes are indicated by arrows. Dashed vertical lines represent the upper and lower limits set for considering transgressive progeny clones

By implementing alcoholic fermentations in a small volume, eleven heritable traits were measured in three grape musts/conditions for 195 strains in duplicate constituting a data set of 14,000 data points (Additional file 2: Table S2). From fermentation kinetics, six representative traits were extracted: lag phase duration (*lp*), time to produce various amount of CO_2_ (*t35 g*, *t50 g* and *t80 g*) and fermentation rate during the first (*V15_50*) and the second part of the fermentation (*V50_80*). Moreover four metabolites present at the end of the fermentation were measured by enzymatic assays: *acetic acid*, *glycerol*, *pyruvate* and *SO*_*2*_. All fermentations were completed since the residual sugars were less than 2 g.L^− 1^. The progress of alcoholic fermentation was measured by estimating the *CO*_*2*_*max* produced, which is stoichiometric to ethanol. The inheritance of the eleven quantitative traits was calculated for each cross and condition (Fig. 3a). Most of the traits were highly heritable since 75% of them had h^2^ values higher than 0.5. Considering all the traits, each cross-by-environment combination had a distinct heritability profile and hierarchical clustering grouped them by environment rather than by cross (Fig. 3a).

The overall level of phenotypic segregation of both sets of segregant was estimated by computing the Euclidean distances within each progeny clone. The trait values for each condition were normalized (center-reduced) for preventing scale effects and only one kinetics trait (*t80 g*) was used, since most of them were strongly correlated (see method) (Fig. 3b). The parental pairwise distances confirmed that SB vs GN are phenotypically more divergent than M2 vs F15 (Euclidian distance = 7.6 and 2, respectively). These contrasting relationships do not impair a wide segregation in both progeny populations. Indeed, the distributions of phenotypic distances are similar between the M2xF15 and SBxGN progenies illustrating that meiotic segregation generated a burst of phenotypic novelty (Fig. 3b). Surprisingly, the phenotypic diversity generated was higher than those observed for the panel of 31 representative commercial starters phenotyped in the same conditions (Additional file 3: Figure S1). As a direct consequence, the percentage of transgressive progenies was much higher in the M2xF15 background, except for *CO*_*2*_*max* and *pyruvate* (Fig. 3c). The percentage of transgression in both crosses is illustrated for the *t80 g* measured in M15_Sk in Fig. 3d. Collectively, these biometric analyses indicate that meiotic segregation generated an important and similar phenotypic diversity despite the contrasting relationships of the two parental pairs. In contrast, the relative phenotypic distance of parental strains had a strong effect on the transgression level observed. In both cases, the phenotypic variability generated significantly exceeded that found for a wide panel of commercial starters. Despite the fact that this analysis was only carried out with two crosses, this underline the power of meiotic segregation to generate phenotypic transgression.

### Distribution of individual reaction norms in segregating populations show ubiquitous GxE

**Fig. 4:**
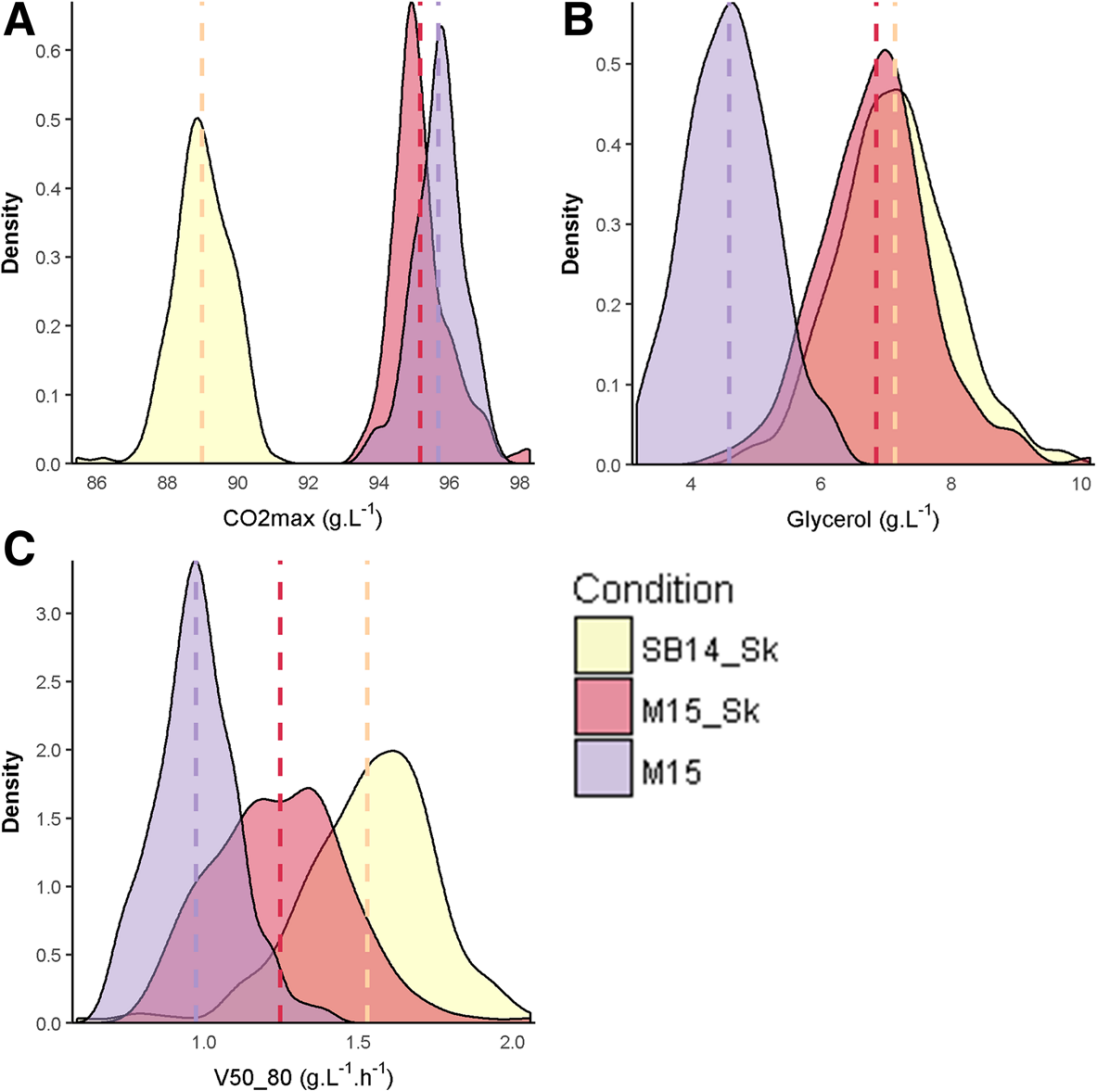
Environnemental impact on quantitative traits Distribution of the phenotypic values according to fermentation conditions. Vertical dashed lines indicate the average value in each condition. *CO*_*2*_*max* is mainly impacted by grape must (Panel **a**), *glycerol* mainly by micro-oxygenation (Panel **b**) and *V50_80* by both (Panel **c**)

The phenotypic plasticity was then analyzed for both progeny populations. First, the impact of the cross was tested using the linear model LM1 that estimates the effects of *cross* (C) and *environment* (E) as well as their primary interaction (C*E) (Additional file 4: Table S3). Analysis of variance shows that the sum of C and C*E had very low effects (below 3.4% in average) for all phenotypes. This result indicates that both crosses have the same phenotypic response to environmental changes. The environmental conditions (μ-Ox and GM) affected the traits in a different manner. Three representative patterns of phenotypic response are shown in Fig. 4; the full details are given in Additional file 5: Table S4. As the two grape musts (SB15 and M14) used have different concentration for SO_2_ (34 mg.L-1 vs 46 mg.L-1) and sugars (194 g.L-1 vs 219 g.L­1), the production of SO_2_ and CO_2_ (Fig. 4a) was directly impacted by the grape juice matrix. In contrast, other traits such as glycerol (Fig. 4b) and acetic acid were mainly impacted by μ-Ox. Indeed, in accordance with previous reports, an increased μ-Ox level increases glycerol production and decreases acetic acid production [50, 53, 56, 59]. Some traits were influenced by both parameters (GM and μ-Ox), including kinetic parameters like *V50_80* (Fig. 4c). The influence of sugar content and μ-Ox on kinetic parameters has also been reported previously [49, 55]. Finally, *pyruvate* production was only slightly impacted by the environmental conditions.

In order to estimate the reaction norm at a strain level, the variation of each trait was decomposed by a second linear model (LM2 see methods). In this model, the overall genetic impact was decomposed into S and S*E components. S represents the constant strain effect across the three conditions (i.e. parallel norm of reaction) whereas S*E represents the phenotypic plasticity among strains (i.e. non-parallel norm of reaction). The analysis of variance estimated the effect of *strain* (S) and *environment* (E) factors as well as the *strain* x *environment* (S*E) interaction (Table 1). The model explained a great part of phenotypic variation (between 62.8 and 96.5% according to the trait). For the majority of traits, environment accounted for most of the variation. However, the constant strain effect S (parallel norm of reaction) also explained an important part of phenotypic variability (up to 52.4% for *pyruvate*). For some traits, an important phenotypic plasticity was observed. This is the case for *lp* (26.1%) and *acetic acid* (20.6%), for which a non-parallel norm of reaction (S*E) made a non-negligible contribution. Interestingly, the genetic weight for CO_2_ production kinetics increased with the fermentation ongoing. Indeed, the sum of S + S*E explained 17.1% of the variance for *t35 g* and linearly increased to 37.6% for *t80* g.

**Table 1:** Analysis of variance of the model LM2 for the 11 phenotypes with 189 strains and three conditions of fermentation

	CO_2_max	Lp	t35 g	t50 g	t80 g	V15_50	V50_80	SO_2_	Acetic acid	Pyruvate	Glycerol
E	90.6 ***	12.8 ***	77.2 ***	71.2 ***	53.3 ***	59.4 ***	53.3 ***	54 ***	22.1 ***	9.2 ***	55.3 ***
S	2.7 ***	23.9 ***	10.8 ***	13.9 ***	21.4 ***	17.8 ***	19.6 ***	18.3 ***	36.1 ***	52.4 ***	13.6 ***
S*E	3.2 ***	26.1	6.3 ***	8.4 ***	16.2 ***	11.7 ***	17.8 ***	12.6 ***	20.6 ***	17.9 **	13.2
Residuals	3.5	37.2	5.6	6.5	9.2	11.2	9.4	15.1	21.2	20.5	17.9

Percentage of variance explained by the LM2 model. Signifiance codes: *p*. val < 0.001 = ***, *p*. val < 0.01 = **, *p*. val < 0.05 = *

**Fig. 5:**
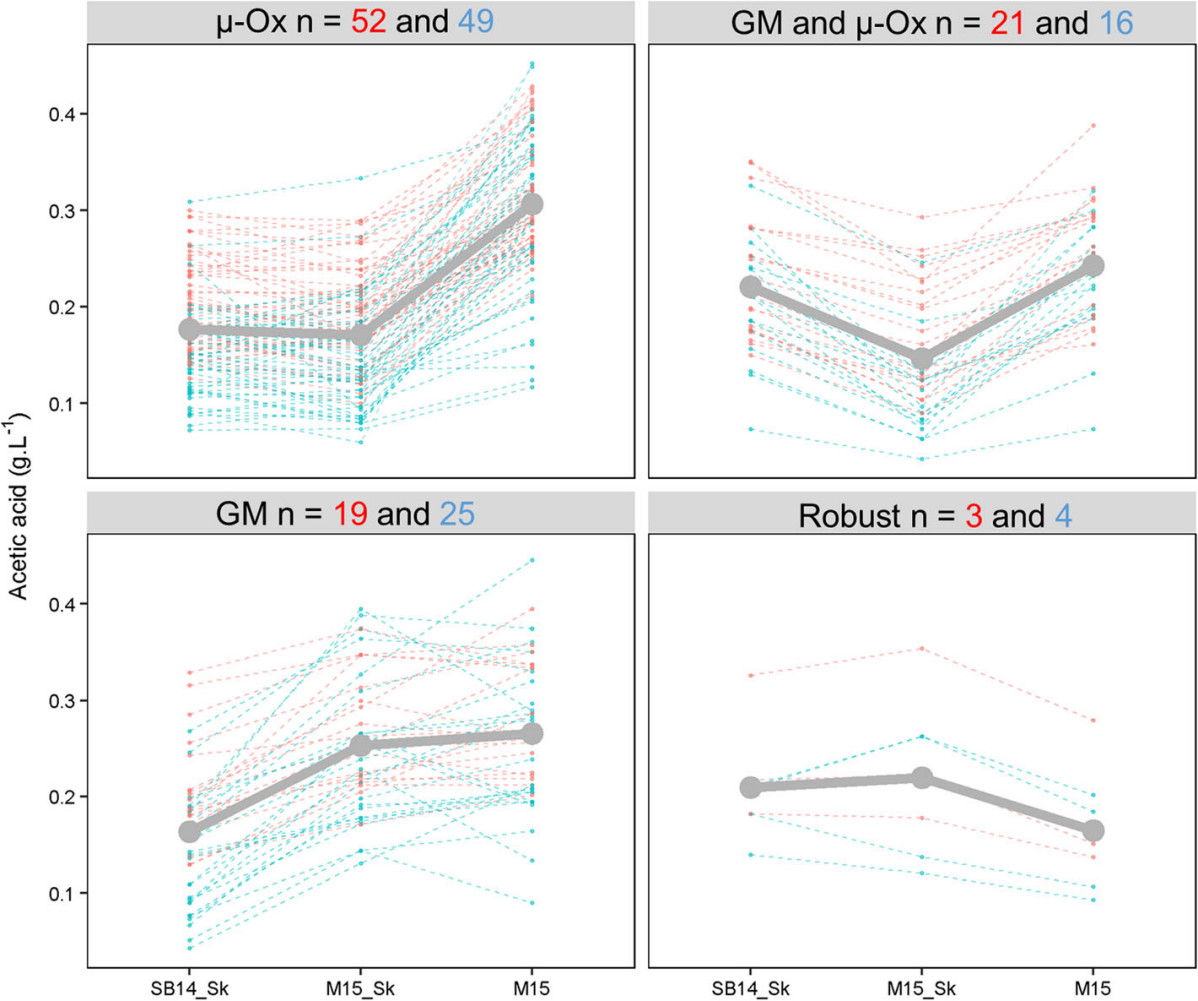
Divergent norm of reaction for acetic acid. Norm of reaction of each individual is shown by dotted line and cluster. They are colored according to the cross (blue for SBxGN cross and red for M2xF15 cross). A grey solid line shows the average norm of reaction of each cluster. Number of strains within each cluster is indicated and colored according to the cross (red for M2xF15 cross and blue for SBxGN cross)

In order to better determine the parallel (S) and non-parallel (S*E) norms of reactions, the phenotypic plasticity of the 189 strains was organized by k-means clustering on the basis of a matrix of correlation distance (see methods). This procedure clustered each progeny clone according to its phenotypic response against environment. Divergent norm of reaction patterns were identified for each trait (Additional file 6: Figure S2). For *acetic acid*, four clusters were obtained, they mainly contained strains with plastic response to micro-oxygenation (μ-Ox: 101 strains), to grape juice (GM: 44 strains), and to the combined effect of GM and μ-Ox (40 strains) (Fig. 5). Strikingly only seven strains (3.7%) appeared robust regarding environmental conditions (Robust cluster). In the four clusters, a similar number of progeny clones was found for the two crosses (chi-squared test, α = 0.05) indicating that the plastic response pattern is not cross specific. From an enological point of view, the magnitude observed is quite relevant since a 0.1 g.L^− 1^ acetic acid difference may impact wine quality [60]. For example, micro-oxygenation had a positive impact for the strains of the cluster μ-Ox and the cluster GM + μ-Ox by significantly decreasing *acetic acid* production (Kruskal-Wallis rank sum test, p val < 0.05). In contrast the strains of cluster GM should be preferred for fermenting white grape juice rather than red matrices (Kruskal-Wallis rank sum test, p val < 0.05).

### Genetic architecture of complex traits for M2xF15 and SBxGN crosses is explained by a similar number of QTLs

**Fig. 6:**
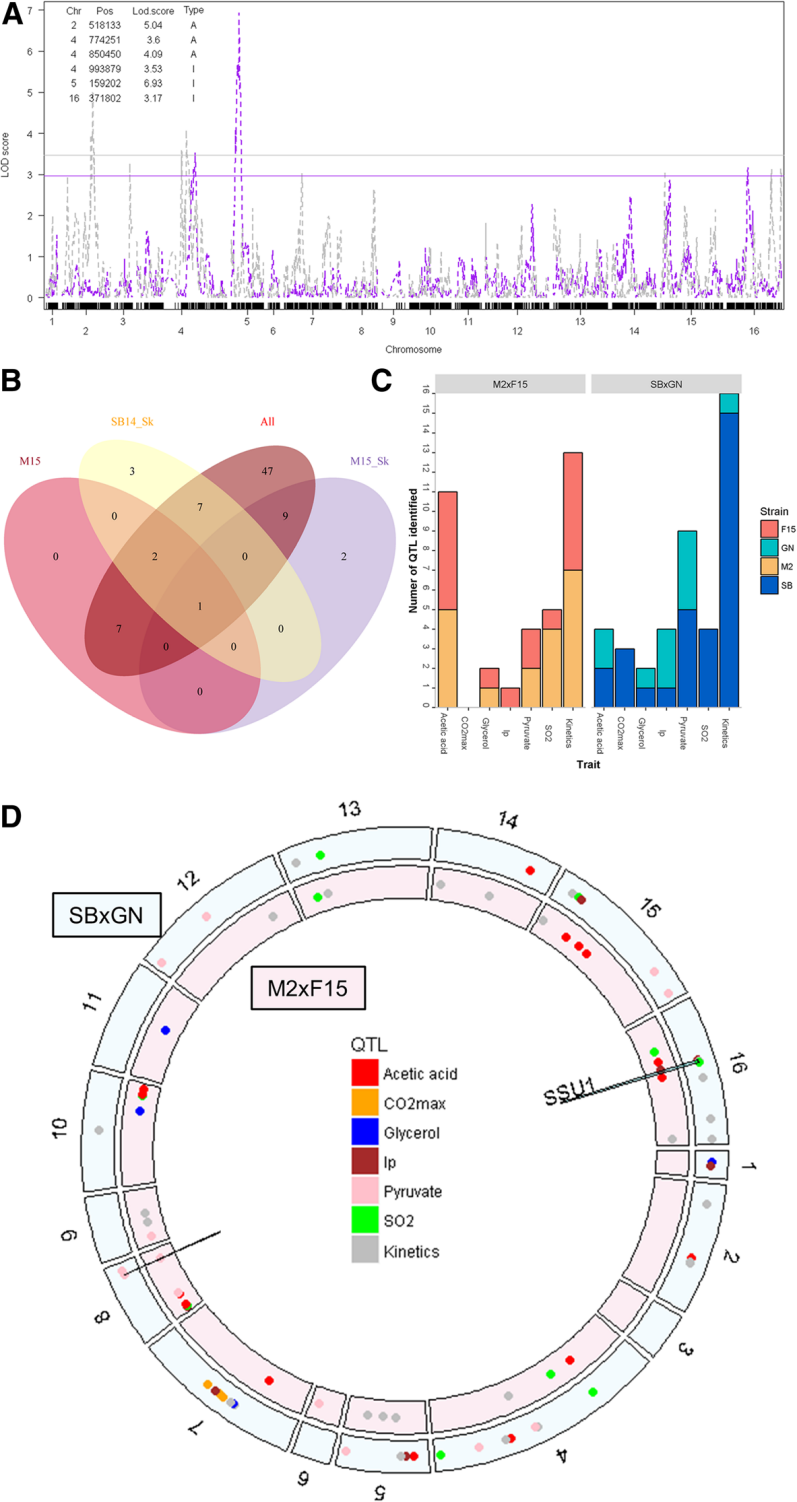
Number of QTLs identified according to cross or environmental conditions. Panel **a**. LOD score obtained with SBxGN cross for *V50_80*. In grey, LOD score for the model with environment as additive variable, in pink, LOD score for the model with environment as interactive variable. The corresponding horizontal colored lines represent the 5% FDR threshold. The table details the QTLs identified by indicating their chromosome (Chr), the position of the marker with the highest LOD score within the QTL peak (Pos), the maximum LOD score within the QTL peak (Lod.score) and the model that detected the QTL (Type, A = additive, I = interactive). Panel **b**. The Venn diagram presents the number of QTLs identified according to the model used. The *All* ellipse corresponds to the multi-environmental model while M15, SB14_Sk and M15_Sk ellipses counts QTLs detected only using a single condition. Panel **c**. The bar chart presents QTLs identified according to cross and trait. Common QTLs identified for *t35 g*, *t50 g*, *t80 g*, *V15_50* and *V50_80* are pooled in Kinetics category and are counted only as one QTL. QTLs are colored according to the parental strains that possess the favorable allele in an enological context. Panel **d**. Distribution of the QTLs identified along the genome according to the cross (M2xF15 inner track, SBxGN outer track). Each point indicates a QTL and is colored according to the trait. The two QTLs that colocalize in the two crosses are indicated with a black pike

The genetic determinism of the eleven traits was elucidated by QTL mapping in both crosses. Phenotypes were linked to segregating genetic markers identified by whole genome sequencing ([61] and this work). The high marker density (0.3 and 0.6 markers/kb for M2xF15 and SBxGN, respectively) ensure a precise localization of QTLs [40, 62, 63]. Interval mapping was carried out by applying a Haley-Knott regression model. This model estimates the effect of each *QTL* detected, the effect of each *Environment* (SB14_Sk, M15, M15-Sk) and the interaction effect between *QTL* and *Environment* (Fig. 6a). Statistical thresholds (false discovery rate (FDR) = 5%) were estimated by 1000 permutation tests [64]. Since the fermentation kinetics traits were partially correlated (*t35 g*, *t50 g*, *t80 g*, *V15_50* and *V50_80*), we found numerous QTLs corresponding to closely related markers. In such cases, a unique QTL was considered in a window of 10 kb and was assigned to the kinetic trait showing the lowest *p* value (see methods). For ease of discussion, all the QTLs found for fermentation kinetics traits were then grouped into the “Kinetics” category. We mapped 78 unique QTLs in the two crosses (Additional file 7: Table S5). With a 5% FDR only less than 4 of these QTLs are expected to be false positives. The efficiency of the multi-environment model was compared to the simplest models, in which only one environmental condition was used. The multi-environment model strongly increased detection power since 45 additional QTLs were detected by this method (Fig. 6b). Five QTLs were only detected with one-environment model and not with the multi-environment model. Rather than additional QTLs, they should be miss localized QTLs as discussed in the discussion. The number of QTLs detected ranged from three (for *CO*_*2*_*max*) to 28 (for *Kinetics*) illustrating the complex genetic determinism of the traits investigated. The number of QTLs identified is correlated with the heritability measured for the trait (Spearman’s correlation coefficient = 0.7, α = 0.05) (Additional file 8: Figure S3).

Despite the contrasting F1-hybrids used (Figs. 2 and 3), a similar number of QTLs was mapped in both progeny populations, with 36 and 42 QTLs for M2xF15 and SBxGN, respectively (Fig. 6c). The positive contribution of each parental strain was evaluated considering the suitable trait value expected according to enological practices. The numbers of positive alleles inherited from M2, F15, SB and GN was 19, 17, 31 and 11, respectively. According to the trait and the cross, the positive alleles were inherited from both or mostly one parent. A noteworthy unbalance was found for kinetics traits since 15/16 of the positive alleles were inherited from the fastest parental strain (SB) in the SBxGN cross whereas only 6/13 were inherited from the fastest parental stain (F15) in the M2xF15 cross (Fig. 6c). In order to find QTLs common to both progeny populations, we sought QTLs impacting the same trait in a 20-kb window (Fig. 6d). Only two QTLs were shared by the two progeny populations. The first locus was detected for *SO*_*2*_, *lp* and *V50_80* and is closely linked to the gene *SSU1* (markers: XVI__SBxGN__371802 and XVI__M2xF15__355235). In the SBxGN progeny, this QTL peak was strongly linked with the marker XV__SBXGN__172951 (Additional file 9: Figure S4). This genetic linkage between two presumably independent markers is caused by a reciprocal translocation event previously described in wine yeasts [65]. The linkage between *SSU1* and the SO_2_ content at the end of the alcoholic fermentation is consistent with its molecular function. Indeed this gene encodes a sulfite transporter (Ssu1p) able to pump out the SO_2_ accumulated in the cytoplasm [66]. The GN inheritance of this QTL, which reduces lag phase by increasing the *SSU1* expression [65] also increases the final SO_2_ concentration (Fig. 6). Interestingly, in the M2xF15 hybrid the marker XVI__M2xF15__355235 was strongly linked to the marker VIII__M2xF15__6499 (Additional file 9: Figure S4). These two loci correspond to a translocation event (VIII-t-XVI) involving the gene *SSU1* previously described by Perez Ortin et al. [67] and present in M2xF15 hybrid [61].

The second common QTL mapped concerns the production of pyruvate and is located on chromosome VIII (markers VIII__SBxGN__446336 and VIII__M2xF15__449469). This QTL explains 19.6 and 30% of the total variance of pyruvate production in M2xF15 and SBxGN cross, respectively. Until now, the sequence analysis of the four parental strains did not reveal any relevant candidate SNP close to this QTL.

All together these results suggest that most of the mapped QTLs are background dependent. Moreover, the similar number of QTLs in both progenies demonstrates that the mapping efficiency is not related, neither to the genetic, nor to the phenotypic distances between parental strains. However, a balanced contribution is more frequent when the strains are phenotypically similar.

### QTLxEnvironment interactions shape the phenotypic variability

**Fig. 7:**
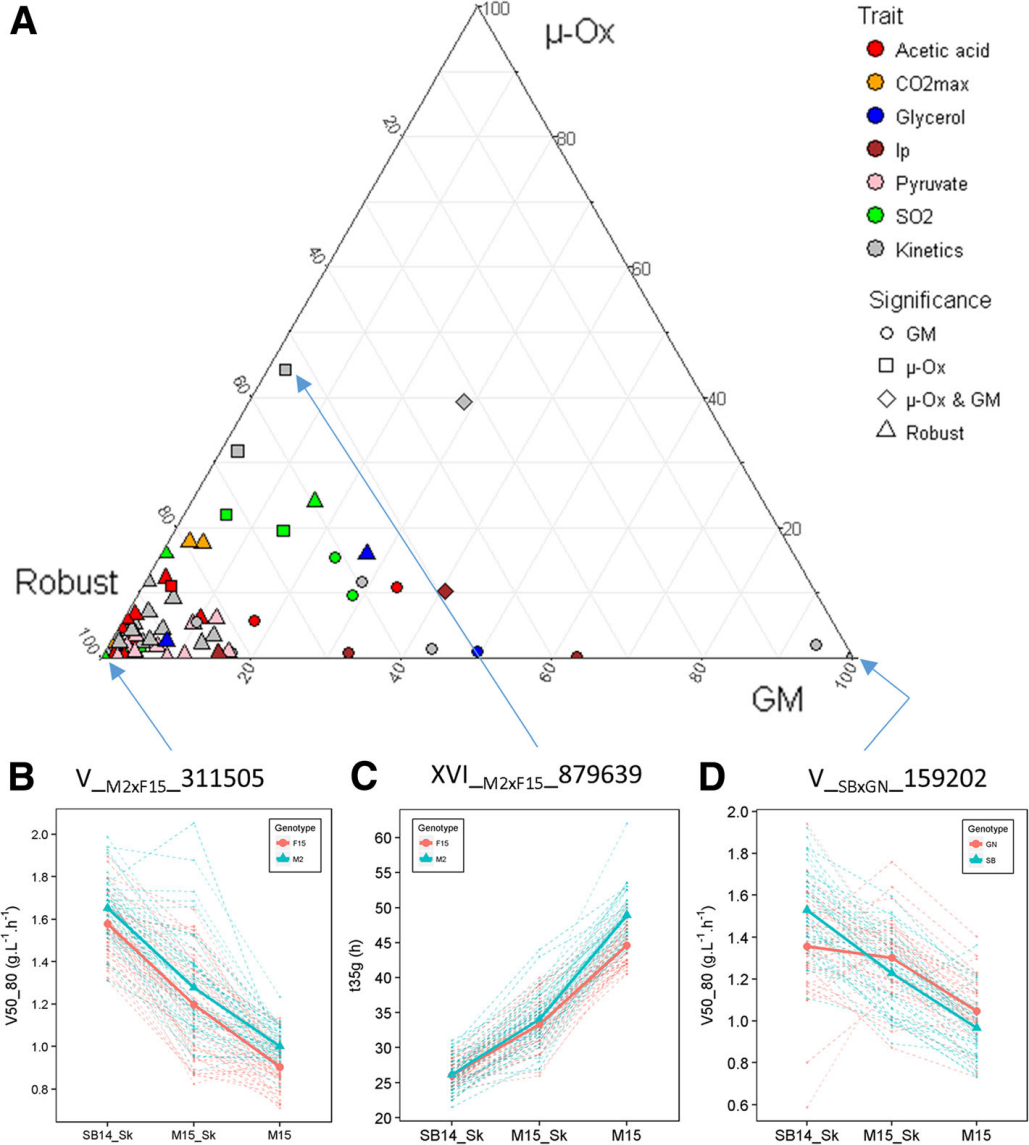
Interaction level of QTLs and environmental conditions. Panel **a**. The ternary plot shows the proportion between genetic effect (G), interaction with grape must (GM) and interaction with micro-oxygenation (μ-Ox) for each QTL. Significance levels were assessed by ANOVA (α = 0.05). Panel **b**. Three examples of QTLs for which extreme levels of interaction were identified (i.e. the less interacting QTL, the most interacting QTL with micro-oxygenation and the most interacting QTL with grape must). Dashed lines show the reaction norm of each segregant according to their allele inheritance. Full lines show average value of the all the segregant according to marker inheritance

The genetic determinism of phenotypic plasticity was then investigated at a genomic scale using the linear model LM3 (see method). For each QTL mapped, the constant genetic effect across environment (G), the interaction effect with grape must (GM), as well as the interaction effect with micro-oxygenation (μ-Ox), were estimated by analysis of variance. From this analysis, QTLs could be sorted as robust (no interaction) or interactive (significant interactions with GM and/or μ-Ox). The overall *GxE* pattern was shown for both crosses (Fig. 7). The less the QTLs interacted with GM and μ-Ox, the closer they were from the bottom left corner of the ternary plot. Most of the QTLs (57/78) were quite robust to environmental changes (Additional file 10: Table S6). This was the case for the QTL V__M2F15__311505 that is one of the most robust. Indeed, progeny clones with both M2 and F15 inheritance showed parallel norms of reaction for *V50_80.* In contrast, 21 *GxE* interactions were detected and most of them (17) were due to the grape must composition. Interactions were mostly a “scale effect” or specific effect as shown for the inheritance of the marker XVI__SBxGN__879639, which had an impact on *t35 g* only in unshaken conditions (Fig. 7). Few loci had antagonistic effects like the QTL V__SBxGN__161933 that influences fermentation kinetics V50_80 according to the grape must. The Additional file 10: Table S6 and Additional file 11: Figure S5 provides a complete overview of *GxE* for the 78 QTLs detected in this work.

### Molecular dissection of the XV-t-VXI translocation demonstrates its interaction with environment

**Fig. 8:**
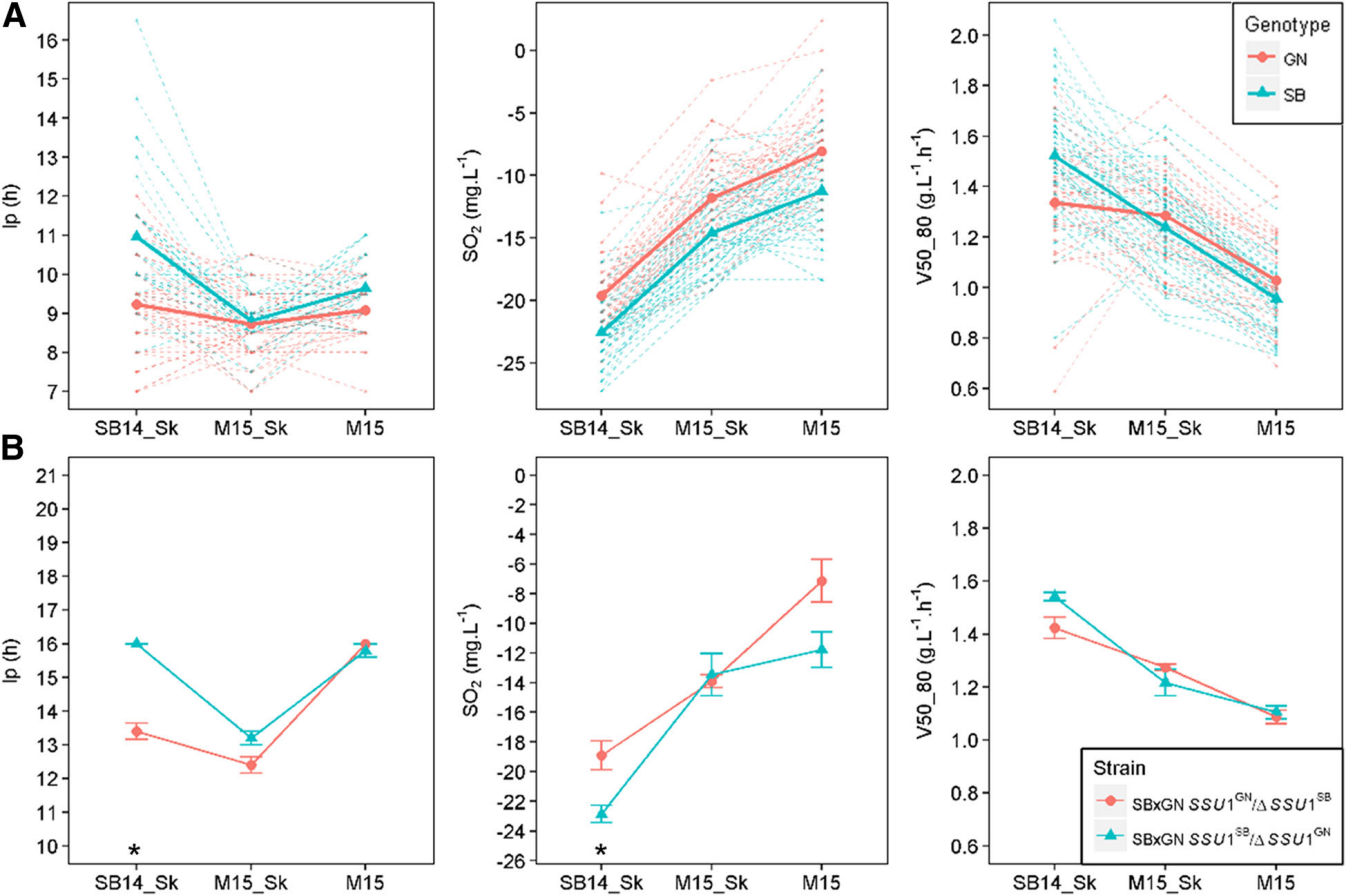
Effect of the XV-t-VXI translocation on phenotypic plasticity. Panel **a**. The norm of reaction of the segregants that inherited from SB or GN for QTL XVI__SBxGN__373847. Dashed lines show the reaction norm of each segregant according to their allele inheritance. Full lines show average value for all segregants according to marker inheritance. Panel **b**. The norm of reaction of the hemizygotes. A star means significant difference (Wilcoxon, α = 0.05)

The accuracy of the QTL mapping and the powerful molecular genetics of *S. cerevisiae* offer the possibility to bridge the gap between a QTL and the causative nucleotide variations. A particular GxE interaction is discussed in this section. Among the QTLs of interactions, XVI__SBxGN__373847 strongly linked three traits (*lp*, *SO*_*2*_, and *V50_80*) and is located in the gene *SSU1*. Since this QTL was physically linked to the marker XV__SBXGN__172951 we concluded that the XV-t-VXI translocation could play a pleiotropic role during alcoholic fermentation. In addition, this QTL showed important *GxE* interactions with GM that are likely due to the difference in the SO_2_ effect between red and white grape juices. In order to test if the *SSU1* inheritance has both pleiotropic and GxE effects, we compared the phenotypic response of previously obtained hemizygous hybrids. Those hybrids are isogenic to SBxGN but have only one functional copy of *SSU1.* The allele *SSU1*^*SB*^ is located in a wild type chromosomal environment (XVI-wt), while the allele *SSU1*^*GN*^ has a translocated environment. The chromosomal break point occurs at the position 161,342 and 373,561 for the chromosome XV and XVI, respectively. This increases its expression due to the proximity of the *ADH1* promoter [65]. The phenotypic responses of hemizygous hybrids were measured in the three conditions (Additional file 12: Table S7) and compared to the two groups of SBxGN according to their inheritance for the *SSU1* locus (Fig. 8). For each of the traits investigated, the progenies reaction norms and hemizygous hybrid responses were very similar. Both interacted significantly with GM and the slope on variation was oriented in the same direction. Strains that have an active *SSU1* translocated gene (hemizygote SBxGN *SSU1*^GN^ / Δ*SSU1*^SB^) or segregants that inherit from GN) are different from other strains with no *lp* increase in SB14_Sk and a 10% lower *V50_80* in SB14. This result demonstrates that *SSU1* allele inheritance accounted for a significant part of the non-parallel reaction norm shown by the SBxGN progeny. Moreover it demonstrates the pleiotropic effect of the *SSU1* gene that impacts lag phase duration but also unrelated phenotypes such as end-product concentration of SO_2_ as well as the fermentation kinetics.

## Discussion

This work aimed to estimate and identify the genetic determinism of phenotypic plasticity. In the last decades, this universal phenomenon has moved from a marginal interest to a new paradigm shaping the evolution of living species in their biotopes [1–3]. Although of great interest, molecular examples of natural genetic variations having a relevant GxE effect are quite scarce [37, 39, 40]. To challenge this task, we used the species *S. cerevisiae* as it is a powerful tool for achieving quantitative genetics [68]. Winemaking conditions allowed us to reproduce a complex and changing environment suitable for identifying alleles conferring phenotypic plasticity. Therefore, in order to match enological practices as closely as possible, the genetic material used was derived from commercial wine starters and the culture conditions used were natural grape juices. This choice was also motivated by the possibility of using the QTLs detected in further selection programs using molecular markers as previously described [69–71].

### Lesson from QTL mapping with divergent wine parental pairs

Before achieving the QTL analysis, we compared the two population progenies demonstrating that the parental pairs are quite divergent. Indeed, in the SBxGN cross the genetic and phenotypic distance of parental strains was much more higher than in the M2xF15 one. In yeast breeding, the selection of parents is mostly based on their phenotypic values. Indeed for optimizing numerous traits, the common strategy consists of selecting parental strains having extreme and opposite phenotypes in order to combine their allelic set in a unique strain [72]. This rational has been adopted by many authors for achieving QTL mapping programs [40, 73–75]. For the first time, this work compares the efficiency of QTL mapping performed with hybrids resulting from close and distant yeast strains. Answering this question comprehensively would ideally require comparison of several crosses at a variety of genetic and phenotypic distances. However, in a first approach, the comparison of two crosses offers a surprising insight. Indeed, QTL mapping efficiency was similar in both populations since the number of QTLs detected as well the part of variance they explained were very similar (Fig. 6 and Additional file 13: Figure S6). Another noteworthy result is the near absence of QTL co-localization (only two loci for 78 QTL detected). This finding has been previously reported in *S. cerevisiae* where the majority of the QTLs are specific to a single cross-combination [76]. However, the four parental strains used in that study were widely divergent (African or Malaysian forest, sake fermentation (Japan) and European wine). In the present work, we observed similar results with strains derived from the same biotope (wine fermentation) that have been subjected to intensive human selection (commercial starters). This could mean that most yeast strains underwent numerous mutations in different loci affecting the same phenotype, as previously demonstrated for beer yeasts [77]. This observation was supported by the fact that phenotypic segregation in both crosses generated phenotypic variability exceeding those found in a wide panel of commercial starters. This promises the possibility of improving strain performance by tapping into this natural mutation pool present within the population of wine strains. It is important to note that each QTLs has a reduced effect, less than 10% of the explained variance. This is consistent with the infinitesimal model that consider that the phenotype variability of a population is explained by few major QTLs and a high number of minor QTLs [78]. The use of two divergent populations was then beneficial for capturing more genetic variability, multiplying by two the potential number of natural variations to explore. This also underlines the wide number of mutations in the yeast genome that can affect a phenotype. The expressivity (penetrance) of such mutations in different backgrounds should be very low due to underlining epistasis. However, two common QTLs were found suggesting that they could be due to positive ongoing selection as discussed below.

### Phenotypic plasticity is the rule and QTL mapping allows the capture of its genetic determinants

In order to detect QTLs explaining plasticity, eleven quantitative traits were measured in three environments. The conditions applied were chosen for reflecting two relevant enological parameters, the nature of grape must (GM) and the micro-oxygenation (μ-Ox) [56]. We first characterized the reaction norm of all the strains, demonstrating that an important part of observed variance was due to strain x environment interactions (up to 26%, Table 1). The acetic acid patterns measured for 189 strains (Fig. 5) revealed that non-parallel reaction norms are the rule and not the exception. By changing the oxygen input (from 2 mg.L^− 1^ in the unshaken condition to 4 mg.L^− 1^ in the shaken condition) or by changing the strain, a variability was observed for 96.3% of the strains with amplitudes that are relevant for the wine industry. As for QTL detection efficiency, the hybrid genetic background does not seem to govern those patterns. This original result demonstrates that GxE are important in wine fermentation and should be better investigated.

The multi-environment QTL analysis drastically increased the statistical power of QTL detection since most of the QTL (45) were only identified with this model, confirming the efficiency of this strategy as previously described [79–81]. Five QTLs were only identified with a single-environment model. Rather than original QTLs, these peaks seem to be QTLs with ambiguous positions that vary greatly between single and multi-environment analyses. Indeed, in an extended window of 100 kb, a QTL identified with the multi-environmental model was found for the same trait. The use of several environments makes it possible to evaluate QTLs robustness. Here, 72% of the QTLs are statistically robust against environment. Since the applied conditions are very different from an enological point of view (white vs red grape must, hypoxic vs micro-oxygenation), most of these QTLs should be robust for most of the fermentations and are suitable for developing selection programs assisted by molecular markers. The 28% of remaining QTLs had a significant interaction with the environment. Except for two QTLs showing antagonistic effects, the interactions detected were due to “scale effect”. This contrasts with the observation made by [33] where QTLs of interaction mostly had antagonistic effect according to environment or were specific to one environment. However the various media tested in that work were quite divergent since the yeast growth was measured in either fermentable or non-fermentable carbon sources, thus creating drastic physiological switches. In our study, the conditions applied remain restricted to the fermentation creating fine-grained interactions.

This study introduces an approach to bridge the gap between non-parallel reaction norms observed and their underlying genetic causes. Some QTLs with significant GxE effects were matched with the strain reaction norms. Among the nine QTLs with significant GxE effects for kinetic traits, two of them (XV__M2xF15__26284 and XVI__SBxGN__371802) had high interaction levels explaining 3.7% and 5% of the variance, respectively. Their inheritance shaped a large part of the phenotypic plasticity of the *V50_80* in both populations (Fig. 8 and Additional file 14: Figure S7) separating the strains in the distinct reaction norms predicted by the k mean clustering (Additional file 6: Figure S2). When non-parallel reaction norms were more complex like *acetic acid* and *pyruvate*, the few QTLs with significant GxE interactions were not sufficient for explaining the overall phenotypic response observed (Fig. 5). However those loci could be helpful for developing breeding programs focusing on specific applications. For example, some strains do not have the appropriate phenotypic response in the presence of micro-oxygenation by not decreasing their acetic acid production (Fig. 5). By interacting with μ-Ox, the QTLs V__SBxGN__70702 and XIV__SBxGN__623501 explained a part of this phenotypic response. The incorporation of their favorable alleles by a cross-breeding strategy in commercial strains, which show inappropriate response to micro-oxygenation, could be very valuable. Based on our data, their additive effect would theoretically reduce up to 66% of this compound in micro-oxygenated conditions. However, all the phenotypic responses were not explained. Indeed, none of the QTL identified for *acetic acid* explains the phenotypic response of strains of cluster GM and μ-Ox that had an increased production of acetic acid in SB14_Sk compared to M15_Sk (Fig. 5). Similarly for *pyruvate*, no interactive QTL was identified while highly divergent norm of reaction were obtained for this trait. This large number of type of reaction norms may reflect a complex genetic determinism with a high number of interacting loci. The segregation of these factors in less than a hundred individuals does not allow their identification. Therefore, the characterization of the genetic determinants of complex traits such as that of *acetic acid* or *pyruvate* seems to require the study of a larger population.

### The Ssu1p sulfite pump a protein under balanced selection have pleiotropic effects

Within an evolving population subject to selection, the genetic variations are a mix of three types: (i) rare and deleterious alleles resulting from recent mutational events not yet eliminated by selection; (ii) neutral alleles whose frequencies follow the rules of genetic drift; (iii) alleles with intermediate frequencies that are subjected to different counterbalanced selection pressures [82–84]. For this last type of allelic variation, the selection pressure can be balanced because one allele may have a favorable or an unfavorable effect according to the environment in which it is expressed, meeting the QTLs described by [33]. Otherwise, the selection pressure can be balanced because one allele can have a pleiotropic effect on fitness parameters, positively affecting some traits but negatively other ones creating phenotypic trade-offs. Mutations with balanced effects have already been identified in *Arabidopsis thaliana* for water use efficiency [37] or delay of germination [38]. They have been useful for understanding why particular accessions are better adapted to specific environment. In yeast, pleiotropic genes (*MKT1 or IRA2*) that affect unrelated traits like temperature resistance [85, 86] or sporulation efficiency [87] were previously described. The effect of the gene *SSU1* in the context of the translocation XV-t-XVI is an interesting case where a single gene can have pleotropic effects on several phenotypes and interaction with environments.

In *S. cerevisiae*, the sulfite pump *Ssu1p* is required for efficient sulfite efflux [66]. The expression of this transporter can be strongly enhanced by chromosomal translocations that modify the promoter environment of *SSU1*. This enhanced expression confers a higher resistance to the inhibitory effect of SO_*2*_ added to the grape must. Two independent translocations (VIII-t-XVI and XV-t-XVI) have been reported in the literature [65, 67], and are hallmarks of adaptation to winemaking practices [88]. Both translocations confer an important adaptive advantage in respect to indigenous flora by reducing the lag phase duration, however, they are not present in all wine strains and are often present in a heterozygous state [65, 89]. In this work, we demonstrated that both translocations impact the final amount of *SO*_*2*_ and the late fermentation rate. Using hemizygous hybrids previously constructed, we validated the pleotropic effect of *SSU1* in the SBxGN background (Fig. 8). The phenotype of the translocated form is in agreement with the expected increase in *SSU1* expression. Therefore, translocated progeny clones leave more SO_2_ at the end of fermentation. More startling is the translocation effect on the late fermentation rate (*V80_80*). Indeed, in the SB14_Sk conditions (containing more active sulfites), the translocated strain has a slower end-fermentation rate that may be due by the higher SO_2_ concentration still present in the medium. This opposed effect of yeast fitness (short lag phase but lower fermentation rate and viability) constitutes a phenotypic trade-off and suggests that *SSU1* has undergone a balanced selection. Despite the great advantage conferred by a short lag phase (more than 20 h in high sulfite conditions) the non-translocated form of *SSU1* also confers adaptive advantages. Indeed, due to their low SO_2_ efflux, the strains having the non-translocated allele do not have to cope with the toxicity of SO_2_ in the late stages of the fermentation, which improves yeast viability. Moreover the effect of the translocation on lag phase duration is much lower in red grape must (Merlot) creating environmental conditions that preserve this allele from selective pressure. Pleiotropic genes are likely important levers of the complex architecture of quantitative traits and they have been reported in other living organisms. The gene *SSU1,* with its translocated forms, is a good example of pleotropic gene promoting phenotypic trade-offs conditioned by environmental conditions.

## Conclusions

This study confirms that QTL analysis in *S. cerevisiae* is a powerful tool for identifying natural genetic variations that impact complex technological traits. Phenotyping under several environmental conditions increased the detection power compared to other studies with the identification of 78 QTLs. This large number of QTLs found between wine parental strains suggest the presence of a large reserve of untapped genetic variations available to improve industrial strains performance. This approach also allowed us to identify the genetic determinants that explain the contrasting phenotypic responses of industrial strains. Although most of the QTLs were robust to environmental changes, some striking GxE interactions were identified. The effects of *SSU1* allelic variants were explained at the molecular level revealing that the sulfite pump Ssu1p has a strong pleiotropic and plasticity role in wine fermentation. These allelic variations are natural and therefore can be incorporated in a non-GMO way, by approaches such as marker-assisted selection, in commercial wine starter cultures. Thus, QTLs that are robust to environmental variation can be used to improve the overall performance of strains, and QTLs whose effects are dependent on the environment can be used to correct a defective phenotypic response.

## Methods

### Yeast strains and culture media

Strains used in this study belong to the yeast species *Saccharomyces cerevisiae*. The four parental strains (SB, GN, M2, F15) are diploid homothallic monosporic clones derived from wine commercially available starters. They were acquired from their respective company. The strains GN, SB and F15 were derived from VL1, Actiflore BO213, Zymaflore F15 (Laffort, Bordeaux, France), respectively, while M2 was derived from Oenoferm M2 (Lallemand, Blagnac, France). The two populations used to perform QTL mapping were obtained from two F1-hybrids (M2xF15 and SBxGN). The first progeny (95 individuals) was obtained by tetrad dissection of the F1-hybrid M2xF15 generated by Huang et al. [90]. The second progeny (94 individuals) was obtained by tetrad dissection of the F1-hybrid SBxGN (formerly named HO-BN by Marullo et al. [70]). Both crosses clones are homozygous and diploid due to the homothallic nature of the parental strains. All the strains were grown at 28 °C on YPD medium (1% yeast extract, 1% peptone, 2% glucose), solidified with 2% agar when required. Sporulation was triggered by plating fresh cells on potassium acetate medium after three days at 24 °C. The strains were stored long term in YPD with 50% of glycerol at − 80 °C. Construction of the hemizygotes SBxGN_SSU1^SB^ / ΔSSU1^GN^ and SBxGN_SSU1^GN^ / ΔSSU1^GB^ (formerly named G092G and S092S) has been described by Zimmer et al. [65].

### Fermentations

The two grape juices used, Merlot of vintage 2015 (M15) and Sauvignon Blanc of vintage 2014 (SB14), were provided by *Vignobles Ducourt* (Ladaux, France) and stored at − 20 °C. Before fermentation, grape juices were sterilized by membrane filtration (cellulose acetate 0.45 μm Sartorius Stedim Biotech, Aubagne, France). Their main enological characteristics are given in Table 2 Sugar content, assimilable nitrogen, pH, total and free SO_2_ were assayed by the enological analysis laboratory (SARCO, Floirac, France). Malic acid was determined by enzymatic assay [56].

**Table 2:** Grape juices used in the study

Grape must	Code	Sugar content (g.L^−1^)	Assimilable Nitrogen (mg N.L^− 1^)	Malic acid (g.L^− 1^)	pH	Total SO_2_ (mg.L^− 1^)	Free SO_2_ (mg.L^− 1^)
Sauvignon Blanc 2014	SB14	194	157	5.6	3.19	34	7
Merlot 2015	M15	219	99	1.9	3.53	46	33

Fermentations were carried out as previously described by Peltier et al. (2018) [56]. Briefly, fermentations were run at 24 °C in 10 mL screw vials (Fisher Scientific, Hampton, New Hampshire, USA) with 5 mL of grape must. Hypodermic needles (G 26–0.45 × 13 mm, Terumo, Shibuya, Tokyo, Japan) were inserted through the septum for CO_2_ release. Two micro-oxygenation conditions were used by applying or not constant orbital shaking at 175 rpm during the overall fermentation. During this study, three fermentation conditions were used: *SB14* with shaking (*SB14_Sk*), *M15* with shaking (*M15_Sk*) and *M15* without shaking (*M15*).

Fermentation progress was estimated by regularly monitoring regularly the weight loss caused by CO_2_ release using a precision balance. The amount of CO_2_ released over time was modeled by local polynomial regression fitting with the R-loess function setting the span parameter to 0.45. Seven parameters were extracted from the model:

- *lp* (h): the lag phase time observed before to release of CO_2_ at 2 g.L^− 1^;
- *t35 g*, *t50 g* and *t80 g* (h): time (minus *lp*) until 35, 50 and 80 g.L^− 1^ of CO_2_ were released;
- *V15_50* (g.L^− 1^.h^− 1^): average sugar consumption between 15 and 50% of *tCO*_*2*_*max*;
- *V50_80* (g.L^− 1^.h^− 1^): average sugar consumption between 50 and 80% of *tCO*_*2*_*max*;
- *CO*_*2*_*max*: maximal amount of CO_2_ released (g.L^− 1^).

### Metabolic compounds

At the end of the fermentation the concentration of four compounds was measured at the metabolomics platform of Bordeaux (http://metabolome.cgfb.u-bordeaux.fr) by semi-automated enzymatic assays [56]. Four phenotypes were measured: *acetic acid* (g.L^− 1^), *glycerol* (g.L^− 1^), *pyruvate* (mg.L^− 1^) (from the final samples taken from each fermentation) and *SO*_*2*_ *Yield* (mg.L^− 1^) ([*SO*_*2*_] _*final*_ - [*SO*_*2*_] _*initial*_).

Each fermentation was carried out two times for the progeny clones and their respective hybrids and 10 times for each parental strain (M2, F15, SB, GN). The entire data set is given in the Additional file 2: Table S2.

### Genotyping and marker map construction by high throughput sequencing

The procedure used for genotyping the 94 SBxGN progenies was the same as published by Marti-Raga et al. [40]. Briefly, all the 94 diploids progeny clones were genotyped by whole-genome sequencing at a low coverage (3–6 X). DNA libraries were pooled and sequenced with a MiSeq apparatus using the standard kit v2 (Illumina) generating paired-end reads of 2 × 250 bp. Filtering and mapping of all sequencing data was performed using publicly available tools (https://usegalaxy.org). Sequencing data were treated as single reads. The main parameters of filtering and mapping were: read trimming (− 39 bases), Phred quality cut-off (Q = 20), and read mapping (BWA software with default parameters). Once the reads had been mapped, BAM files were extracted and a *pileup* dataset was generated using SAMTools’ [91] for every segregant. The *pileup* dataset was opened in R and SNP between the parental strains was evaluated using an R script [40]. To construct the marker map, we retained the markers with a 1:1 segregation among the progeny (Chi-x test, α > 0.05) and being evenly distributed along the genome (1 marker/15 kb). The final map generated had 3433 markers (Additional file 15: Table S8). The procedure used for genotyping the 95 M2xF15 progenies was described by Roncoroni [61].

### Microsatellite genotyping

The DNA of *S. cerevisiae* strains was quickly extracted in 96-well microplate format using a customized LiAc-SDS protocol [92]. Fifteen polymorphic microsatellite loci SCAAT3 (*C3*, *C5*, *SCYOR267C*, *C8*, *C11*, *SCAAT2*, *YKL172*, *SCAAT6*, *C9*, *C4*, *SCAAT5*, *SCAAT1*, *C6*, *YPL009*, *YKL172W*) were used for estimating the genetic relationships within 96 commercial starters and the four monosporic parental strains (GN, SB, M2, F15) used in this work (Additional file 16: Table S9). The genotyping conditions used were broadly those described by Börlin et al. [93]*.* Briefly, two multiplex PCRs allowing genotyping of seven loci were carried out in a final volume of 12.5 μL containing 6.25 μL of the Qiagen Multiplex PCR master mix and 1 μL of DNA template, and 1.94 μL of each mix was added in the mixture using the concentrations indicated in the Additional file 16: Table S9. Both reactions were run with the following program: initial denaturation at 95 °C for 5 min, followed by 35 cycles of 95 °C for 30 s, 57 °C for 2 min, 72 °C for 1 min, and a final extension at 60 °C for 30 min. The size of PCR products was analyzed by the MWG company (Ebersberg, Germany) using 0.2 μL of 600 LIZ (GeneScan) as a standard marker. Chromatograms were analyzed with the GeneMarker (V2.4.0, Demo) program.

### Data analyses

All the statistical and graphical analyses were carried out using R software [94].

### Estimation of environment, cross and strain effect

The variation of each trait was estimated by the analysis of variance (ANOVA) using the *aovp* function of the *lmPerm* package in which significance of the results were evaluated by permutation tests instead of normal theory tests.

The *LM1* model estimated the effects of the cross, of the environment and of the cross-by-environment interaction of fermentation traits according to the following formula:

$$ {y}_{ij}=m+{C}_i+{E}_j+{\left(C\ast E\right)}_{ij}+{\epsilon}_{ij}. $$

where *y*_*ij*_ was the value of the trait for cross *i* (*i* = 1, 2) in environment *j* (*j* = 1, 2, 3), *m* was the overall mean, *C*_*i*_ was the cross effect, *E*_*j*_ the environment effect, (*C* ∗ *E*)_*ij*_ was the interaction effect between cross and environment and *ϵ*_*ijk*_ the residual error.

The *LM2* model estimated the effects of the strain, of the environment and of the strain-by-environment interaction on fermentation traits according to the following formula:

$$ {y}_{ij}=m+{S}_i+{E}_j+{\left(S\ast E\right)}_{ij}+{\epsilon}_{ij}. $$

where *y*_*ij*_ was the value of the trait for strain *i* (*i* = 1, …, 189) in environment *j* (*j* = 1, 2, 3), *m* was the overall mean, *S*_*i*_ was the strain effect, *E*_*j*_ the environment effect, (*S* ∗ *E*)_*ij*_ was the interaction effect between strain and environment and *ϵ*_*ijk*_ the residual error.

### Estimation of the genetic distances within strains

The microsatellite dataset was manipulated using the *adegenet* package [95] implemented in R. The percentage of missing data was 1.6%. The genetic distance within the strains was estimating using the Bruvo’s distance using the *poppr* package [96]. The unrooted dendrogram was built by Neighbor Joining (*ape* package) [97]. Since the bootstraps estimated did not allow the resolution of clear groups, the genetic structure was estimated by a *k* mean clustering using the function *find.cluster* allowing the detection of three main groups. The minimal goodness of fit was selected using comparing AIC, BIC and WSS criteria using default parameters.

### Estimation of the phenotypic distance within strains

Phenotypic distances were computed by calculating the Euclidian distances within the strains of the same group in each environmental condition and for each trait. For comparison of the overall phenotypic distance between the commercial starters (*n* = 31) (obtained in [56]) and each progeny population (*n* = ~ 100), 1000 random pools of 31 spore clones of each progeny population were compared to the commercial dataset by a one way analysis of variance *aovp* function of *lmPerm* package. Tukey’s honest significant difference post hoc test was used to confirm differences between groups (α = 0.05).

The phenotypic variability of M2xF15 and SBxGN progenies measured in M15 was visualized by a Principal Component Analysis (PCA) using the *ade4* package (Additional file 3: Figure S1). An additional dataset, recently obtained by Peltier et al. [56], was added to the projection corresponding to the phenotypic values of 31 commercial strains. M15 condition was compared with the only similar condition described in that work (noSk.5_SV). The contribution of each phenotypes to the PCA dimensions and the correlation circle were obtained by the *fviz_contrib* and the *fviz_pca_var* functions of the *factoextra* package [98].

### Norm of reaction clustering

For each trait the distance between norms of reaction were calculated according to the formula:

$$ {D}_{ij}=\left|1- corr\left(\left(\begin{array}{c}P{1}_i\\ {}P{2}_i\\ {}P{3}_i\end{array}\right),\left(\begin{array}{c}P{1}_j\\ {}P{2}_j\\ {}P{3}_j\end{array}\right)\right)\right| $$

where *D*_*ij*_ was the distance between strains *i* and *j*, *P*1_*i*_ and *P*1_*j*_ the phenotypic values measured in *SB14_Sk*, *P*2_*i*_ and *P*2_*j*_ the phenotypic values measured in *M15_Sk* and *P*3_*i*_ and *P*3_*j*_ the phenotypic values measured in *M15* for strains *i* and *j*, respectively. The minimal distance (0) was set between norm of reactions with null variances and the maximal distance (1) was set between norm of reaction with a null variance and all the other norm of reactions with a non-null variance. K-means clustering was performed on distance matrix with the *pam* function of the *cluster* package of the R program. Appropriate number of cluster was determined by the best silhouette value between one and ten clusters with the *silhouette* function of the *cluster* package of the R program.

### Estimation of heritability, transgression level and phenotypic distance within segregating population

The *lato* sensu heritability *h*^*2*^ was estimated for each phenotype according to Marullo et al. [72] as follows:

$$ h2=\frac{\sigma {P}^2-{\sigma E}^2}{\sigma {P}^2}. $$

where *σP*^2^ is the variance of progeny population in each environmental conditions, explaining both the genetic and environmental variance of the phenotype measured, whereas *σE*^2^ is the median of the variance of replicates in each environmental conditions, explaining only the environmental fraction of phenotypic variance.

Percentage of transgression was calculated as described in *Marullo* et al. (2006) [72]. Results were displayed on a heatmap (*heatmap.2* function) of the *gplots* package [99].

### QTL mapping

Before linkage analysis phenotypes were normalized by Rank-transformation using the *GenABEL* package [100]. The QTL mapping analysis was performed with the *R/qtl* package [101] on the data collected in the three environmental conditions by using the Haley-Knott regression model that provides a fast approximation of standard interval mapping [102]. Environment effect and its interaction with QTL effect were assessed by adding environment as additive and interactive covariate. As environment has a large effect on the phenotype, its inclusion in the analysis reduce residual variation that is not genetic and therefore enhance QTL detection. It also allows to asses QTL x Environment interactions. For each phenotype, a permutation test of 1000 permutations tested the significance of the LOD score obtained, and a 5% FDR threshold was fixed for determining the presence of QTLs [64]. QTLs identified for the fermentation rate parameters t35 g t50 g t80 g V15_50 and V50_80 being in the same 10 kb windows were considered as a unique locus. The QTL position was estimated as the marker position with the highest LOD score among all markers above the threshold in a 30 kb window.

### Estimation of the level of QTL interaction

The interaction level of each QTL was estimated by ANOVA using the *aovp* function of the *lmPerm* package. The LM3 model estimated the effects of the cross, of the environment and of the QTL-by-environment interaction on traits according to the following formula:

$$ {y}_{ij}=m+{Q}_i+{E}_j+{\left(Q\ast E\right)}_{ij}+{\epsilon}_{ij}. $$

where *y*_*ij*_ was the value of the trait for allele *i* (*i* = 1, 2) in environment *j* (*j* = 1, 2 or *j* = 1, 2, 3), (three combinations of environments were considered: a) SB14_Sk and M15_SK to assess the grape must effect (GM), b) M15_Sk and M15 to assess the micro-oxygenation effect (μ-Ox), c) SB14_Sk, M15_SK and M15 to assess the overall genetic effect), *m* was the overall mean, *Q*_*i*_ was the QTL effect, *E*_*j*_ the environment effect, (*Q* ∗ *E*)_*ij*_ was the interaction effect between QTL and environment and *ϵ*_*ijk*_ the residual error. In order to compare the level of interaction across traits and environment, their % of variation explained was calculated by omitting the total sum square of environment. The ratio between grape must interaction, micro-oxygenation interaction and genetic effect was calculated as the ratio of the percentage of variation explained by QxE by considering SB14_Sk and M15_SK for grape must interaction, M15_Sk and M15 for micro-oxygenation interaction and the percentage of variation explained by Q in all conditions for the overall genetic effect.

## Additional files

Additional file 1: **Table S1.** 97 commercial strains used (XLSX 10 kb)

Additional file 2: **Table S2.** Phenotypic dataset (XLSX 108 kb)

Additional file 3: **Figure S1.** Meiosis emphases phenotypic novelty. Panel A. PCA of winemaking properties of M2xF15 and SBxGN progenies and 31 CWS in M15. Panel B. Correlation circle indicating the correlation of the variables for axes 1 and 2. Panel C. Average phenotypic distance computed from M15 condition for CWS, and the two M2xF15 and SBxGN cross. (PDF 455 kb)

Additional file 4: **Table S3.** LM1 model for the 11 phenotypes with 189 strains and three conditions of fermentation (XLSX 11 kb)

Additional file 5: **Table S4.** Phenotypic plasticity at the population level. The first line indicates M15_Sk average phenotypic values and the two other indicate the effect of each environmental parameter: difference between M15_Sk and SB14_Sk (grape must effect), difference between M15_Sk and M15 (micro-oxygenation effect). Significant differences are indicated by * (Wilcoxon test pval < 0,05). (XLSX 11 kb)

Additional file 6: **Figure S2.** Clustering of Norm of reaction for each trait. Norm of reaction of each individual is shown in dotted line and are colored and faceted according their cluster. Solid line shown the average norm of reaction of each cluster. Number of strains within each cluster is indicated by n. (PDF 2768 kb)

Additional file 7: **Table S5.** List of QTL identified. (XLSX 17 kb)

Additional file 8: **Figure S3.** Heritability is correlated to the number of QTLs detected. The data represented are the number of QTLs identified according by cross and by trait according to the average heritability by cross and by trait among the three conditions. (PDF 21 kb)

Additional file 9: **Figure S4.** Linkage between translocation markers in M2xF15 and SBxGN crosses. The data represented are the number of segregant according to their genotype for the marker next to the chromosomal break point. Genotypes A strong linkage is shown with less than 10% recombinants. (PDF 4 kb)

Additional file 10: **Table S6.** Number of QTL and their interaction level. (XLSX 10 kb)

Additional file 11: **Figure S5.** Variation of QTL effect according to condition. For each QTL, the values shown are the difference between the phenotypic values measured for all the segregant that inherited the allele of SB or M2 minus those that inherited from GN or F15. A star means a significant difference (Wilcoxon, α = 0.05). (PDF 25 kb)

Additional file 12: **Table S7.** Hemizygote dataset. (XLSX 13 kb)

Additional file 13: **Figure S6.** Variance explained by QTL according to cross. Each dot represent a QTL. Bigger points indicate average. There is no significant difference between the two cross (Wilcoxon test pval > 0,05). (PDF 721 kb)

Additional file 14: **Figure S7.** Impact of QTL XV__M2xF15__26284 on *V50_80.* Panel A. The norm of reaction of the segregants that inherited from M2 or F15 for QTL XV__M2xF15__26284. Dashed lines show the reaction norm of each segregant according to their allele inheritance. Full lines show average value of the all the segregant according to marker inheritance. Panel B. Enrichment of strains having inherited the F15 allele in cluster 2. Norm of reaction of each individual is shown in dotted line and faceted according to their cluster. M2xF15 progeny clones are colored according to their allele inheritance at XV__M2xF15__26284 (red for F15 and blue for M2). A grey solid line shown the average norm of reaction of each cluster. Number of M2xF15 progeny clones within each cluster is indicated by n=; the first number indicate the number of strains from that inherit from M2 and the second one from strain that inherit from F15. A star means a disequilibrium in the theoretical homogeneous distribution 0.5 / 0.5 chis square test, α = 0.05). Panel C. Coverage difference between M2 and F15 strains within genomic region of QTL XV__M2xF15__26284. (PDF 154 kb)

Additional file 15: **Table S8.** Genetic map of SBxGN progeny. (XLSX 2066 kb)

Additional file 16: Primers information and reaction condition for microsatellite genotyping. (XLSX 12 kb)

## Abbreviations

ANOVA: Analysis of variance
C: Cross effect
C*E: Cross-by-environment interactions
CWS: Commercial Wine Starters
E: Environement Effect
FDR: False discovery rate
G: Genetic effect
GM: Grape must effect
GxE: Gene-by-environment interactions
LM1: Linear model 1
LM2: Linear model 2
lp: lag phase
M15_Sk: Merlot 2015
M15_Sk: Shaken Merlot 2015
Name: Abbreviations
PCA: Principal Component Analysis
S*E: Strain-by-environment effect
SB14: Sauvignon Blanc 2014
SB14_Sk: Shaken Sauvignonc Blanc 2014
SO2: Sulfur dioxide
μ-Ox: micro oxygenation effect
