# Supplementary figures and images for: Dissection of the molecular bases of genotype x environment interactions: a study of phenotypic plasticity of Saccharomyces cerevisiae in grape juices
Source: BMC Genomics. 2018 Nov 9;19:772. doi: 10.1186/s12864-018-5145-4 (PMC6225642; doi:10.1186/s12864-018-5145-4)

A

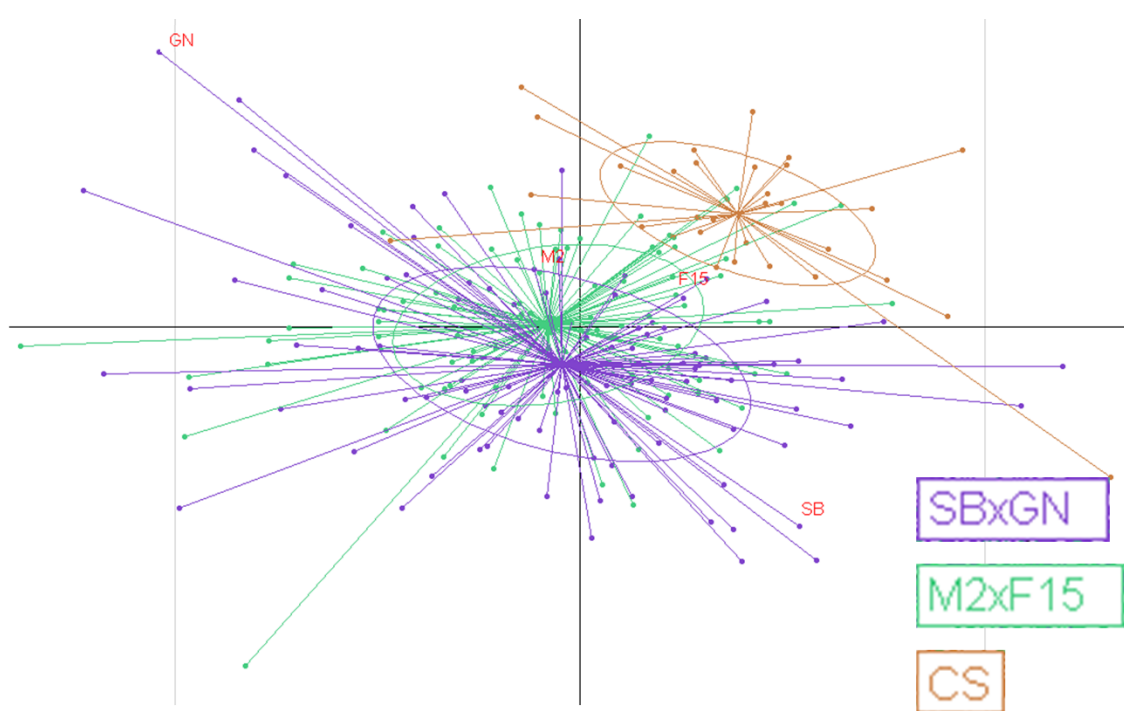

B

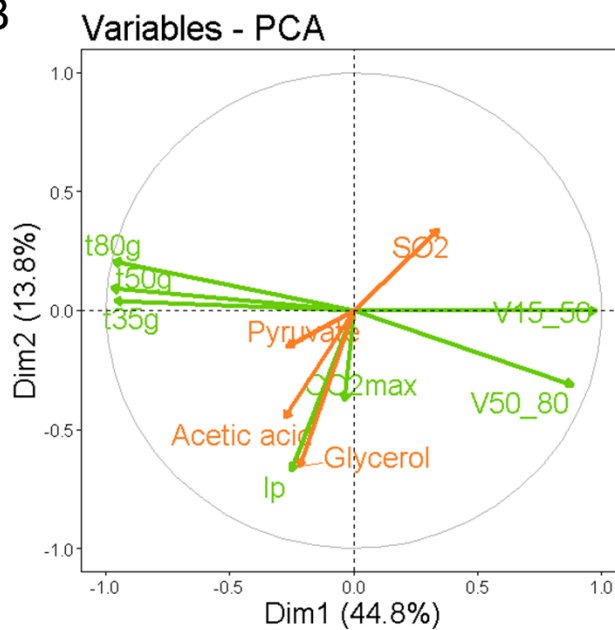

C

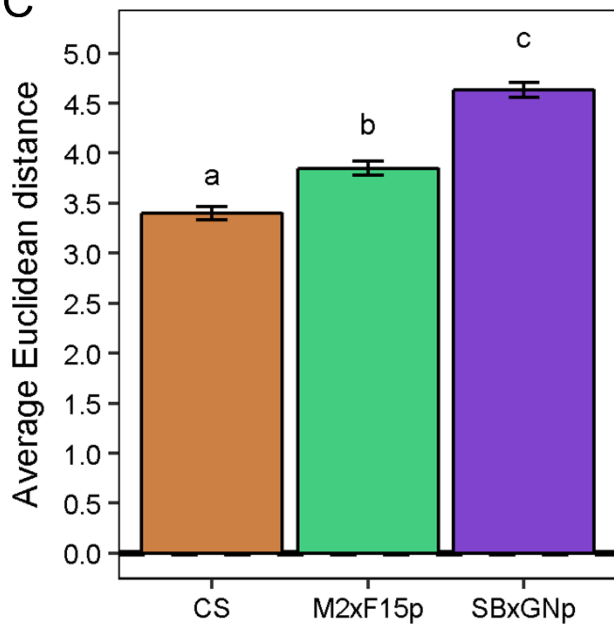

Supplement: Supplementary file 3 — Figure S1. Meiosis emphases phenotypic novelty. Panel A. PCA of winemaking properties of M2xF15 and SBxGN progenies and 31 CWS in M15. Panel B. Correlation circle indicating the correlation of the variables for axes 1 and 2. Panel C. Average phenotypic distance computed from M15 condition for CWS, and the two M2xF15 and SBxGN cross. (PDF 455 kb) [file 12864_2018_5145_MOESM3_ESM.pdf]

Correlation coefficient (spearman) = 0.7 (pval = 0 )

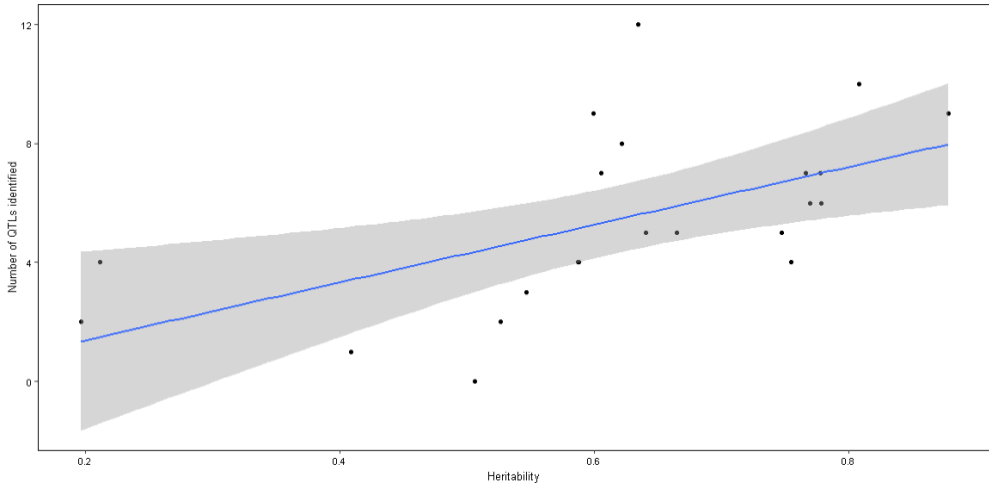

Supplement: Supplementary file 8 — Figure S3. Heritability is correlated to the number of QTLs detected. The data represented are the number of QTLs identified according by cross and by trait according to the average heritability by cross and by trait among the three conditions. (PDF 21 kb) [file 12864_2018_5145_MOESM8_ESM.pdf]

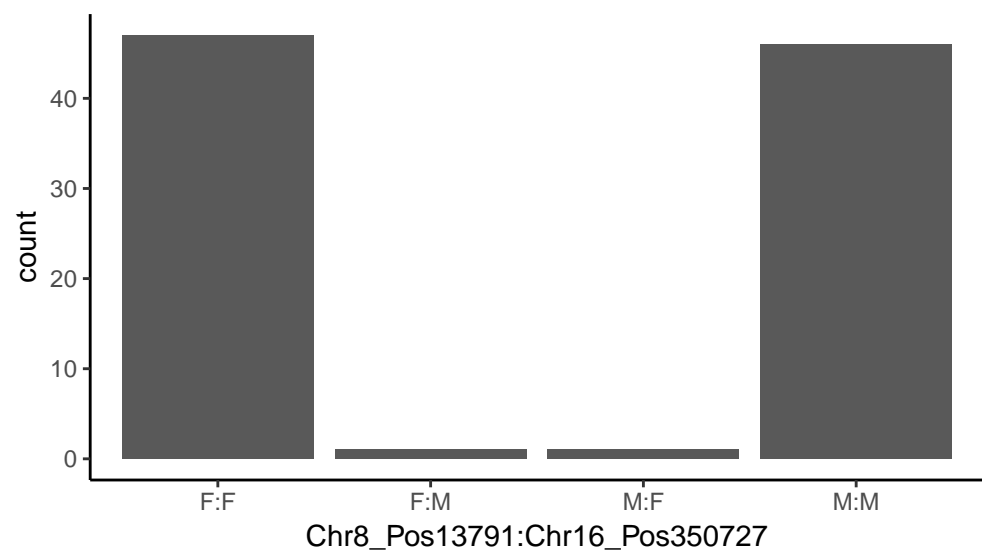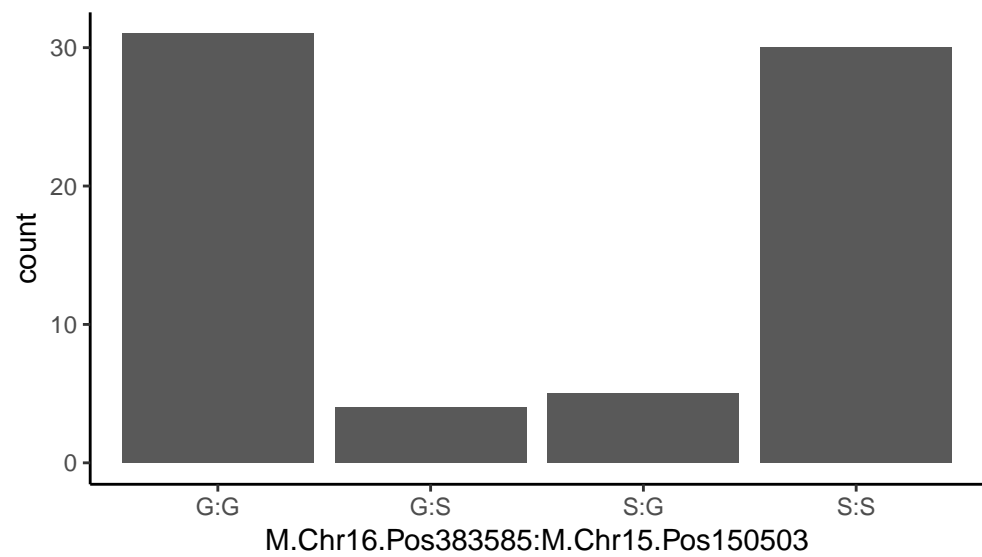

Supplement: Supplementary file 9 — Figure S4. Linkage between translocation markers in M2xF15 and SBxGN crosses. The data represented are the number of segregant according to their genotype for the marker next to the chromosomal break point. Genotypes A strong linkage is shown with less than 10% recombinants. (PDF 4 kb) [file 12864_2018_5145_MOESM9_ESM.pdf]

% of var explained

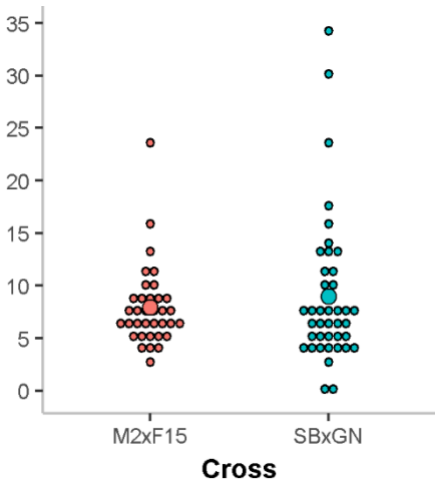

Supplement: Supplementary file 11 — Figure S5. Variation of QTL effect according to condition. For each QTL, the values shown are the difference between the phenotypic values measured for all the segregant that inherited the allele of SB or M2 minus those that inherited from GN or F15. A star means a significant difference (Wilcoxon, α = 0.05). (PDF 25 kb) [file 12864_2018_5145_MOESM11_ESM.pdf]

# XV\_M2xF15\_26284

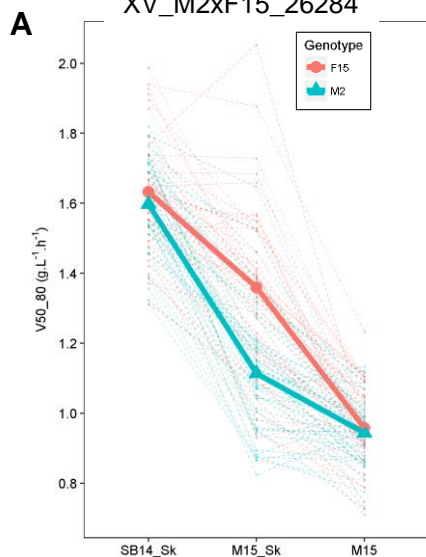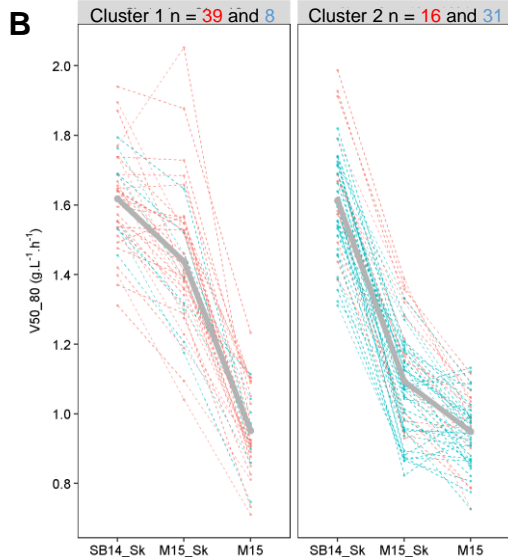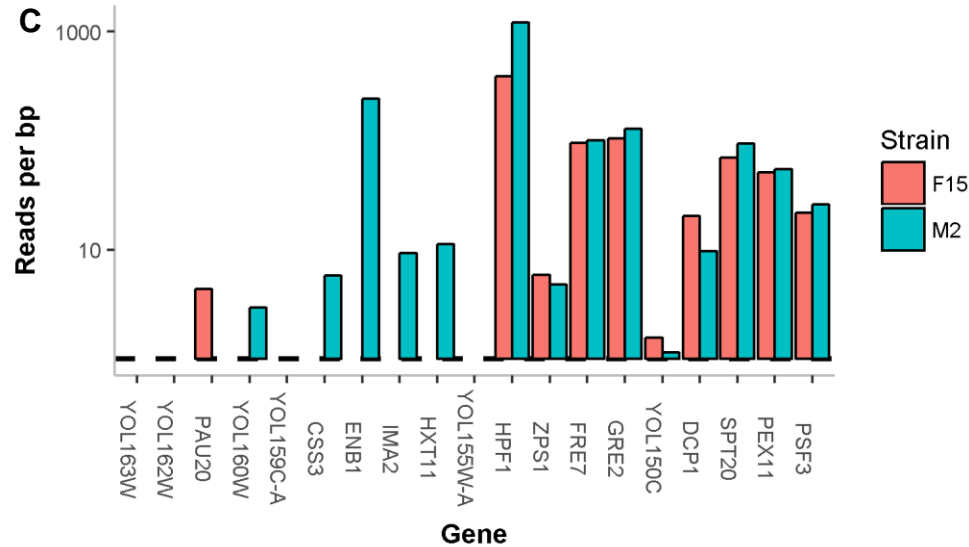

Supplement: Supplementary file 13 — Figure S6. Variance explained by QTL according to cross. Each dot represent a QTL. Bigger points indicate average. There is no significant difference between the two cross (Wilcoxon test pval > 0,05). (PDF 721 kb) [file 12864_2018_5145_MOESM13_ESM.pdf]

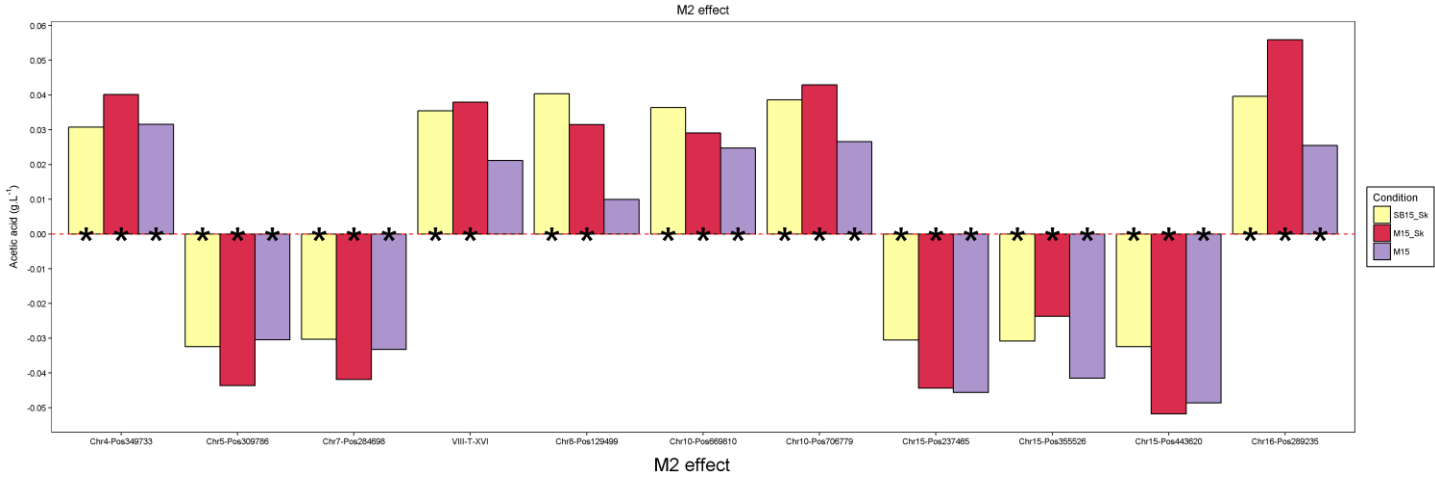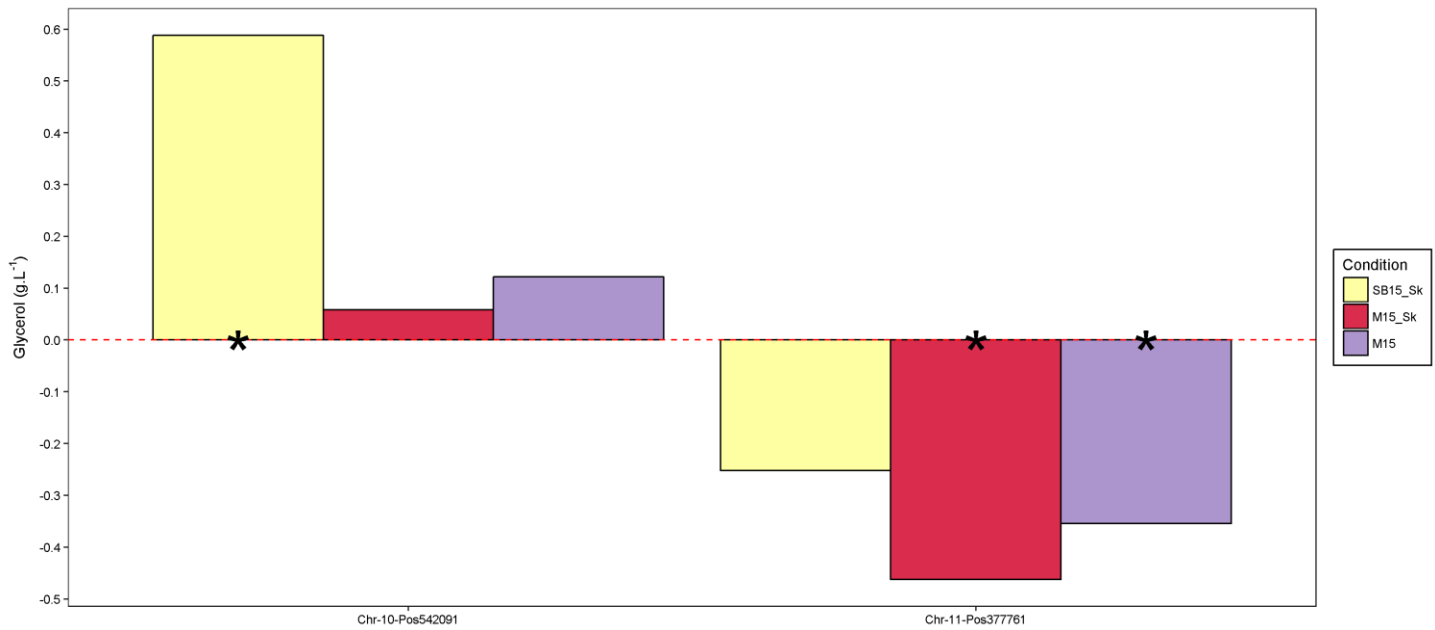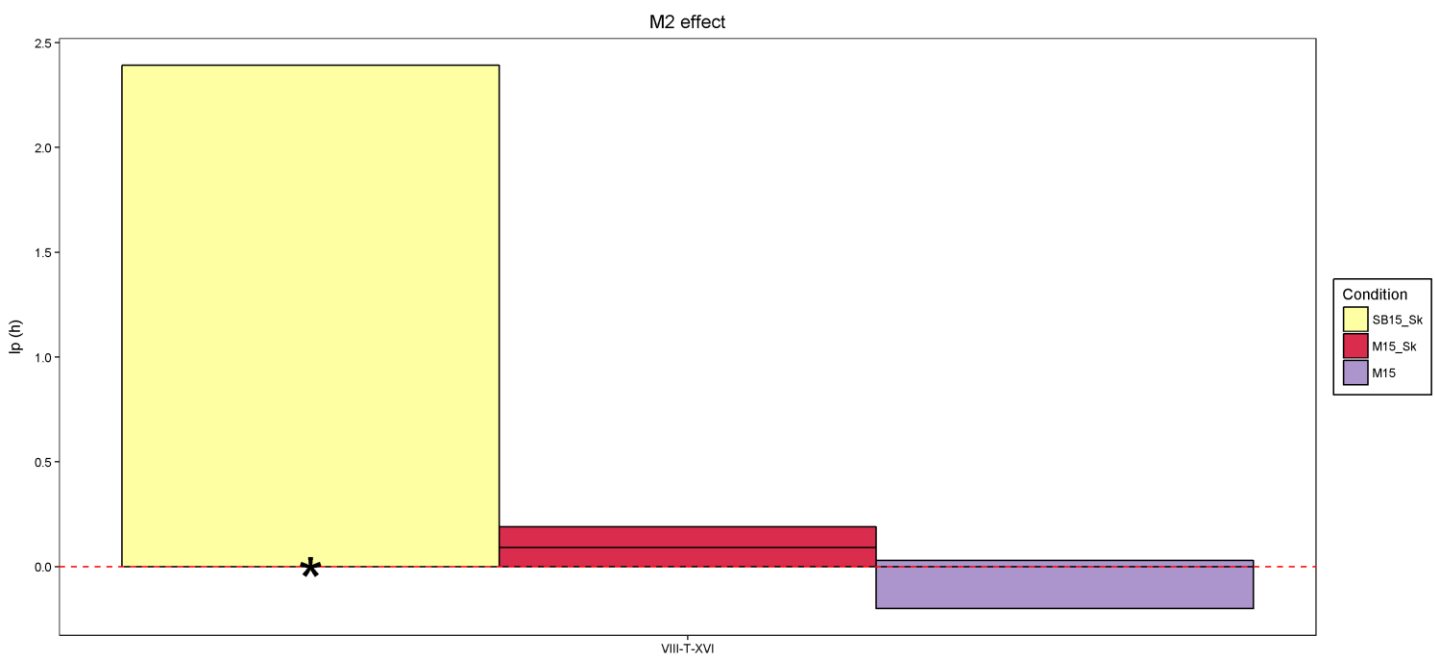

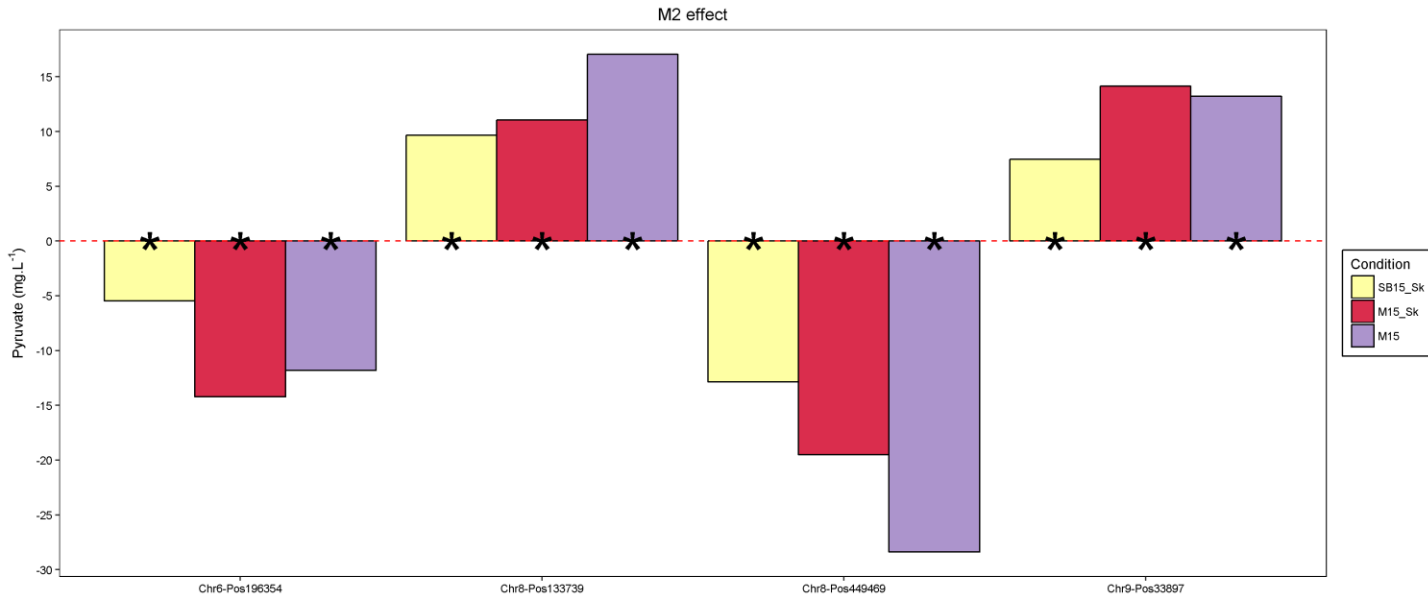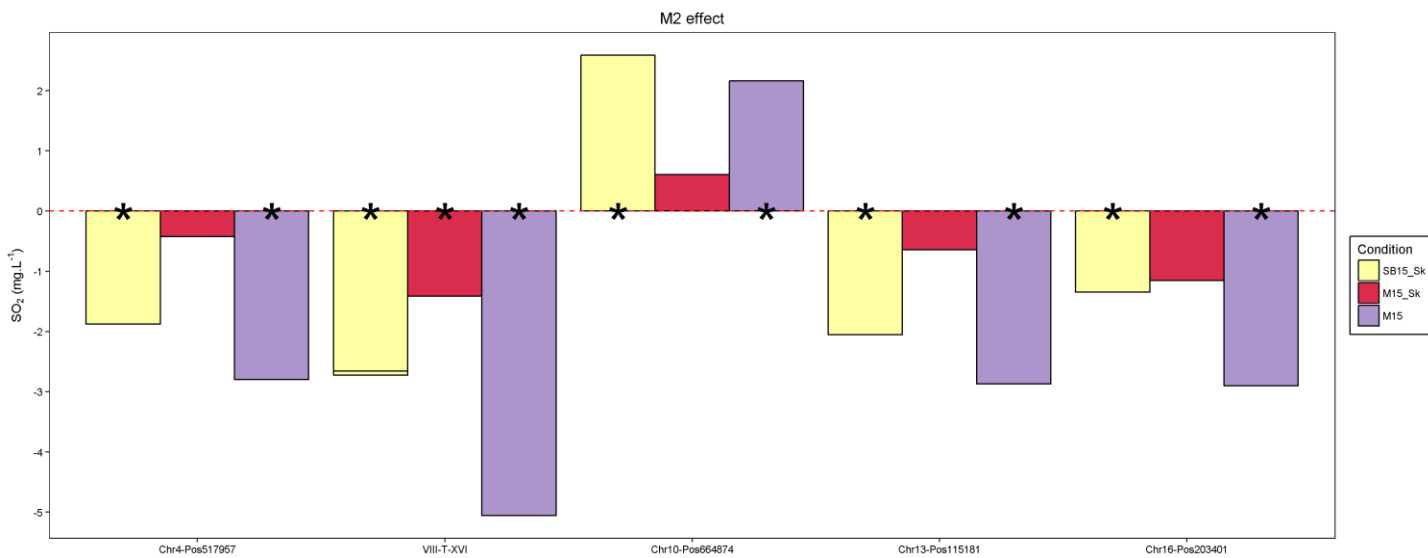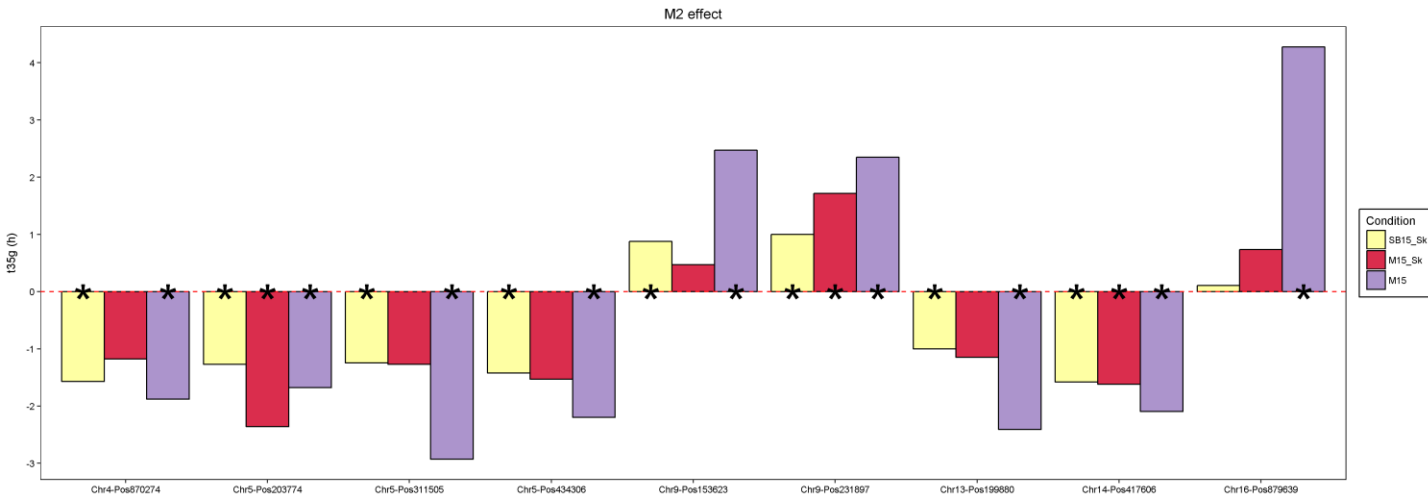

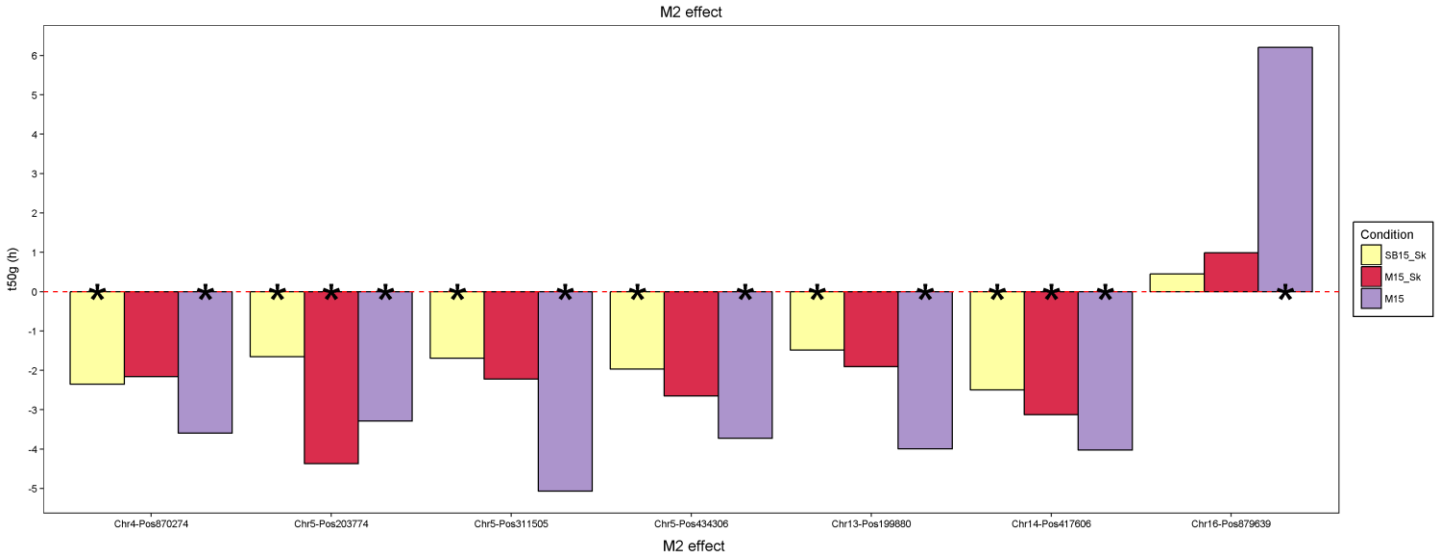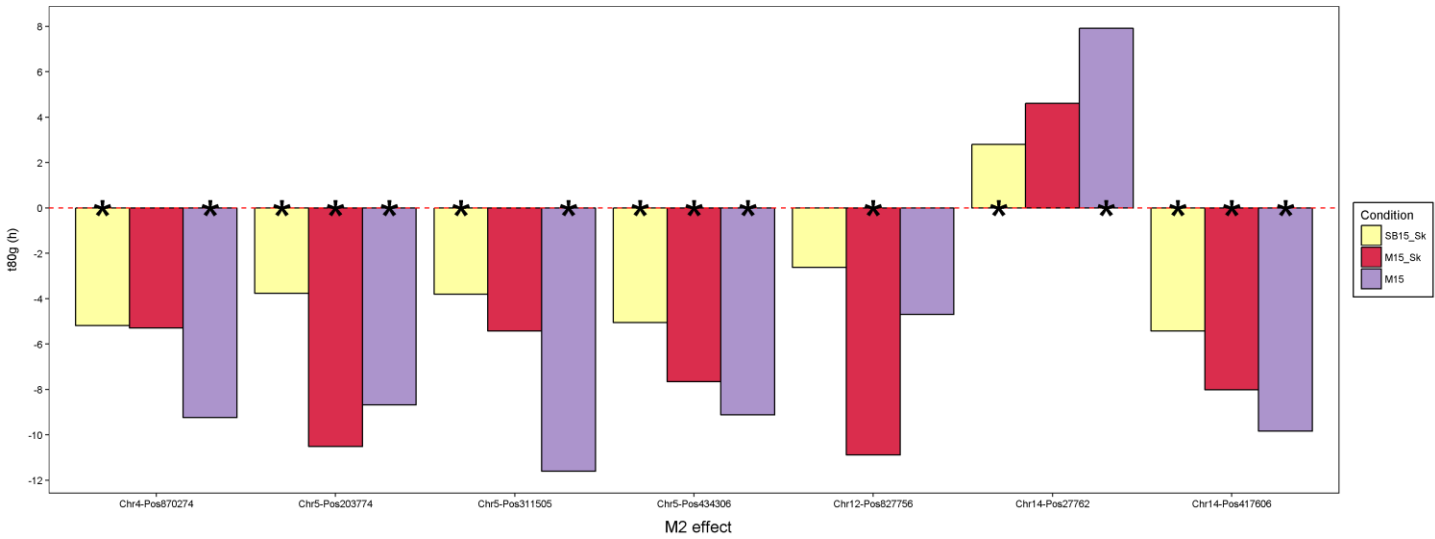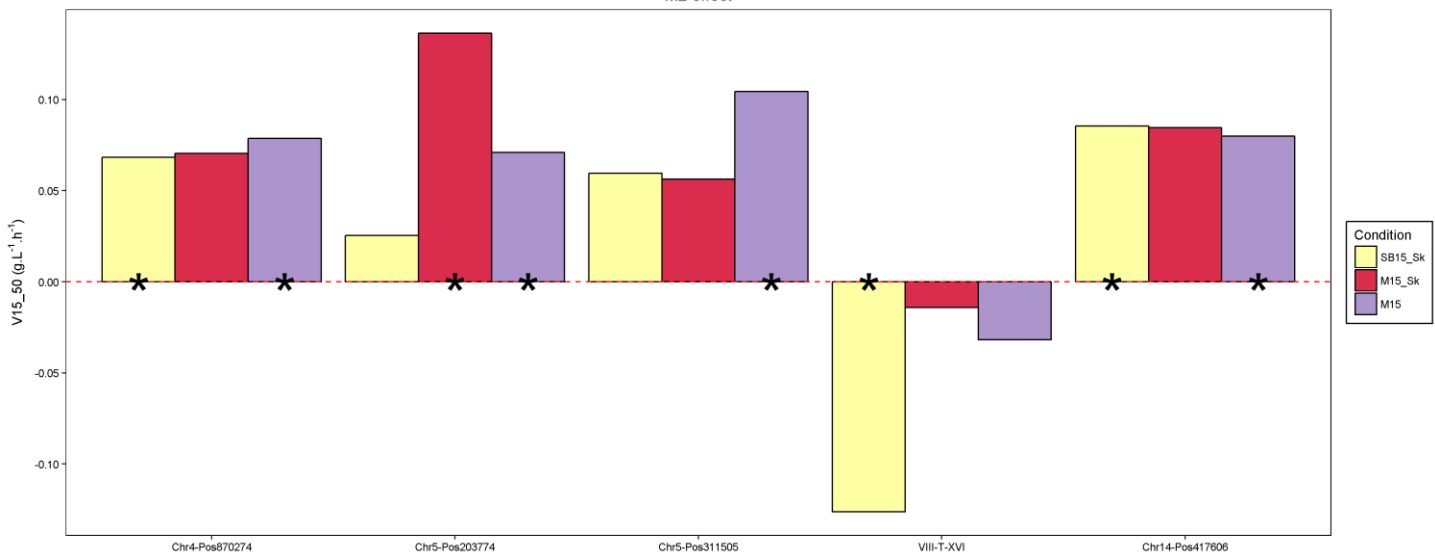

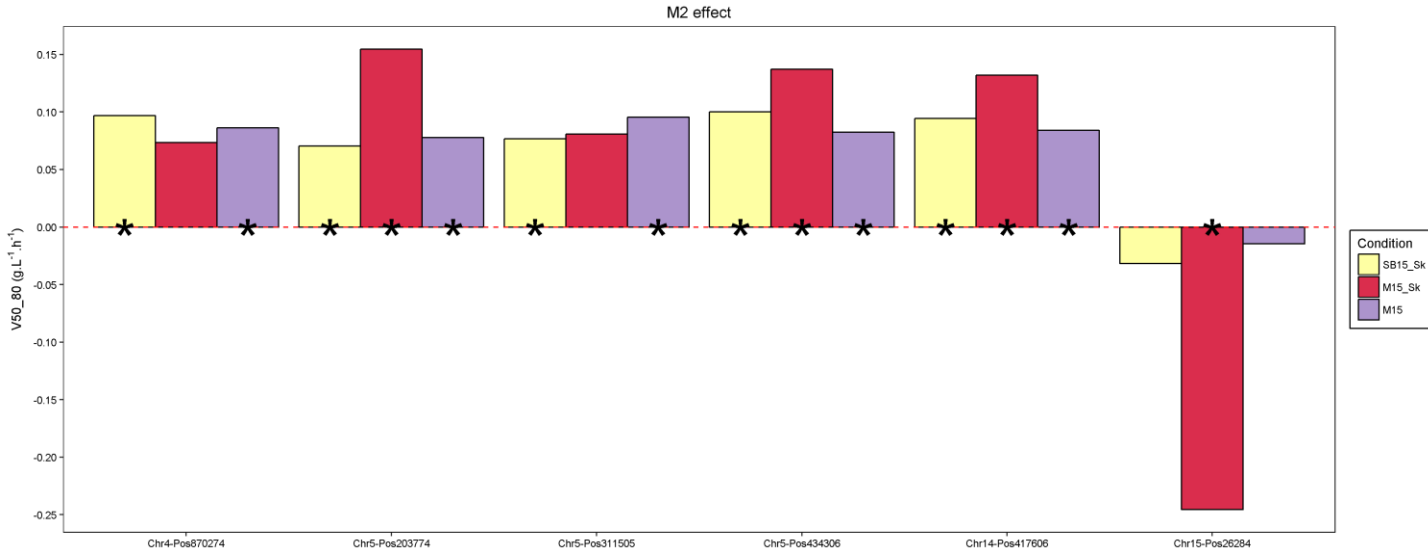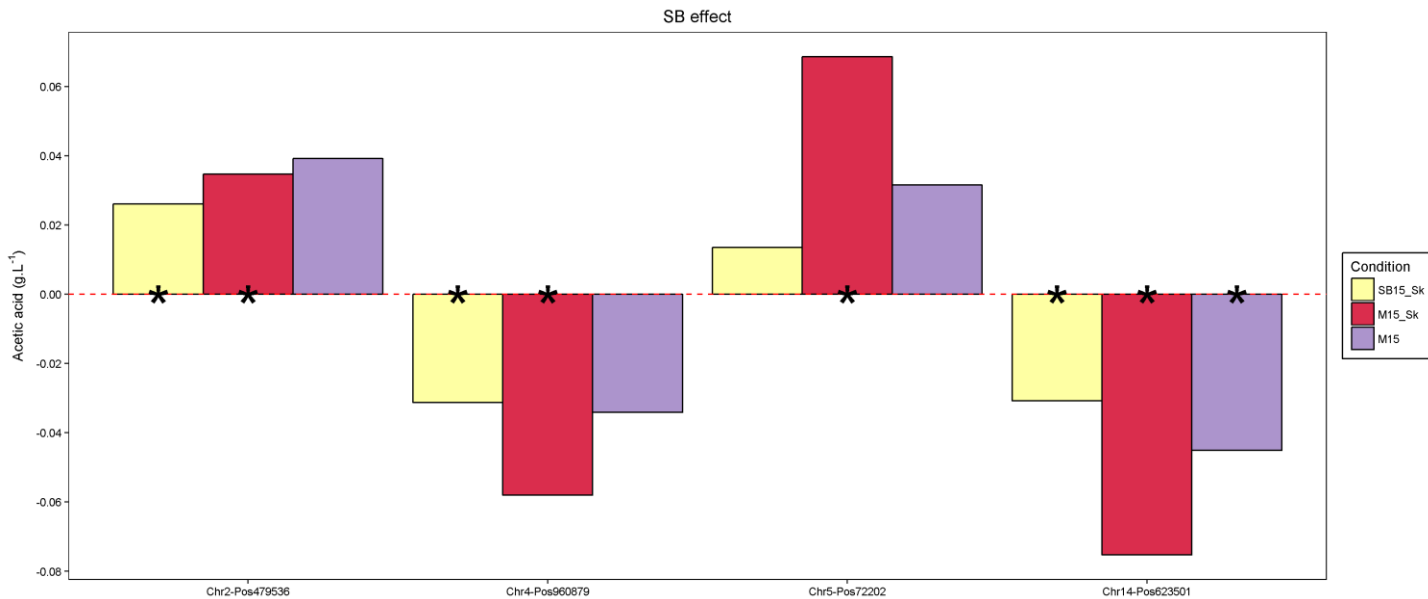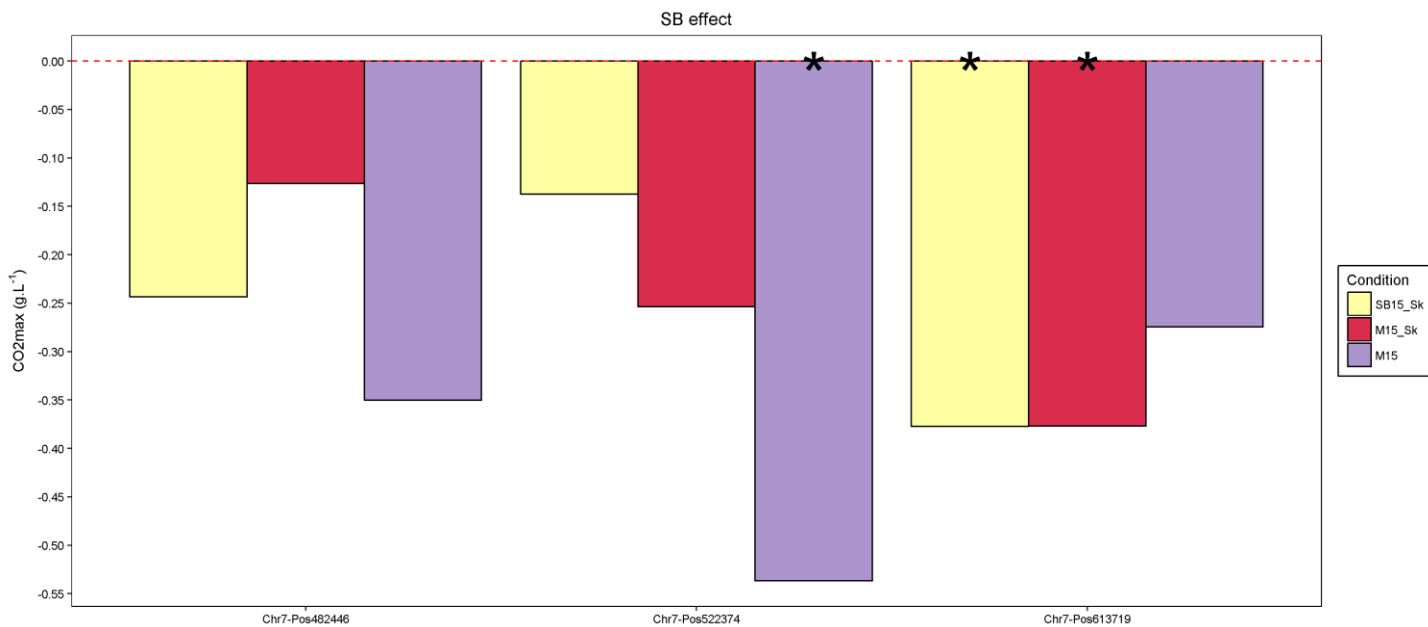

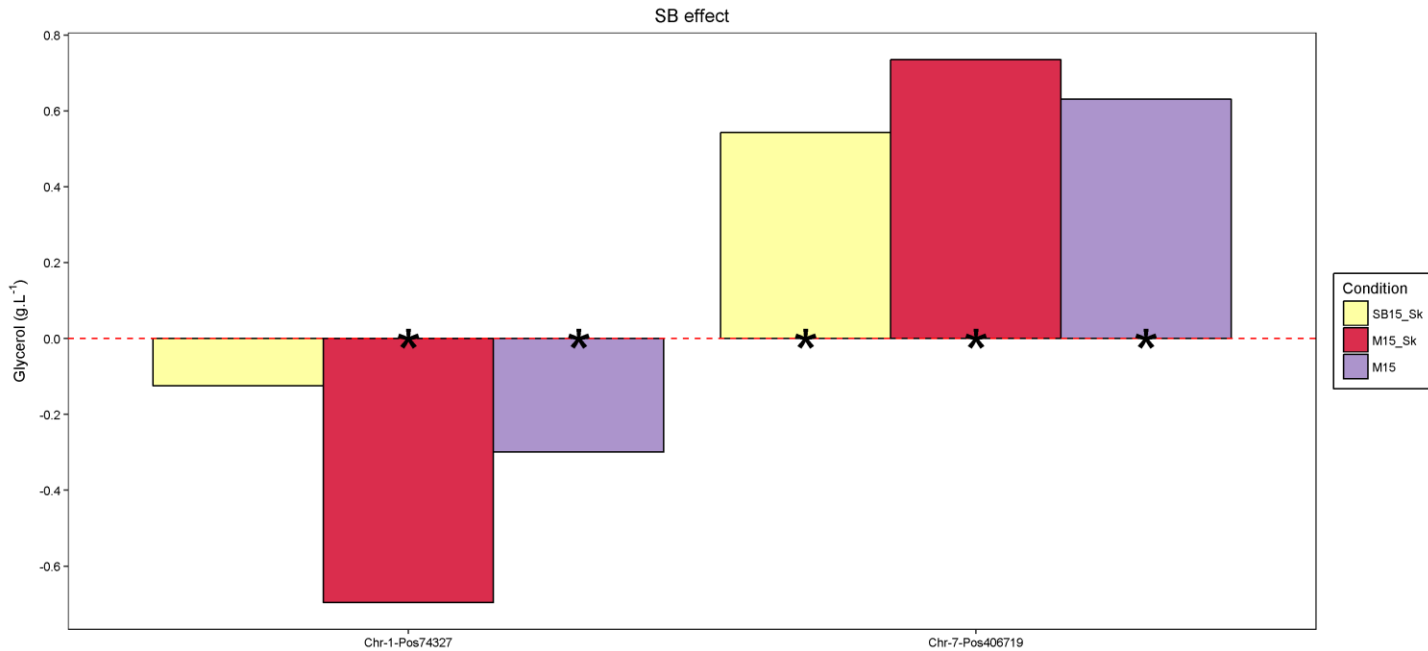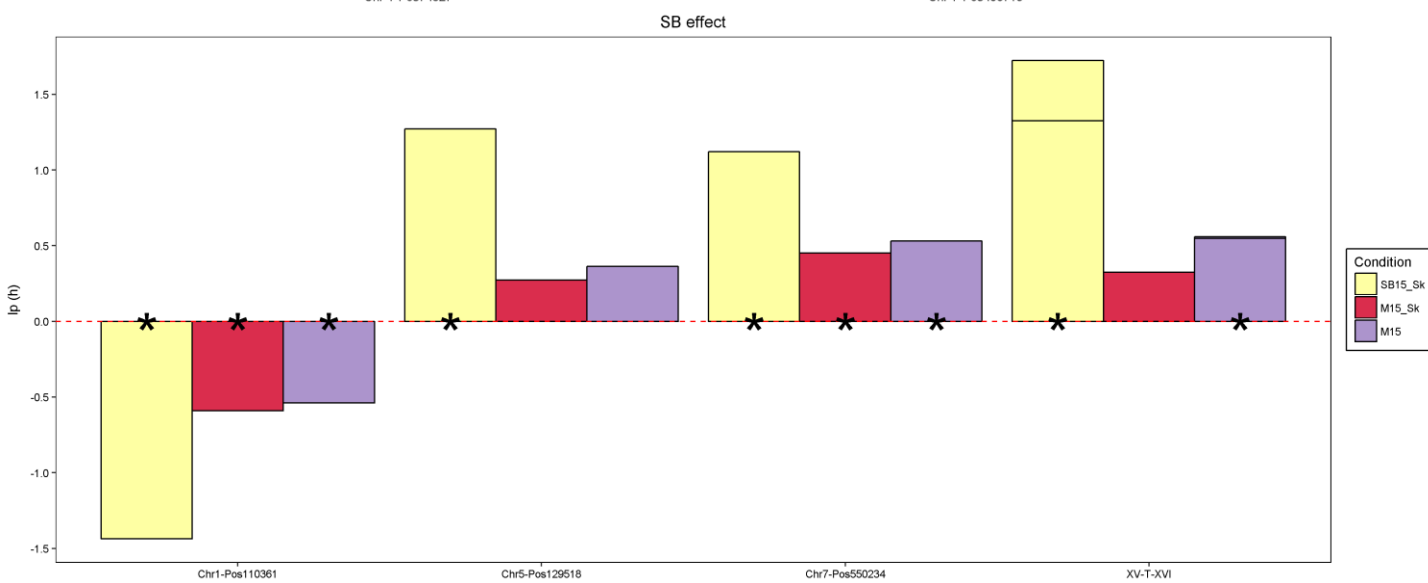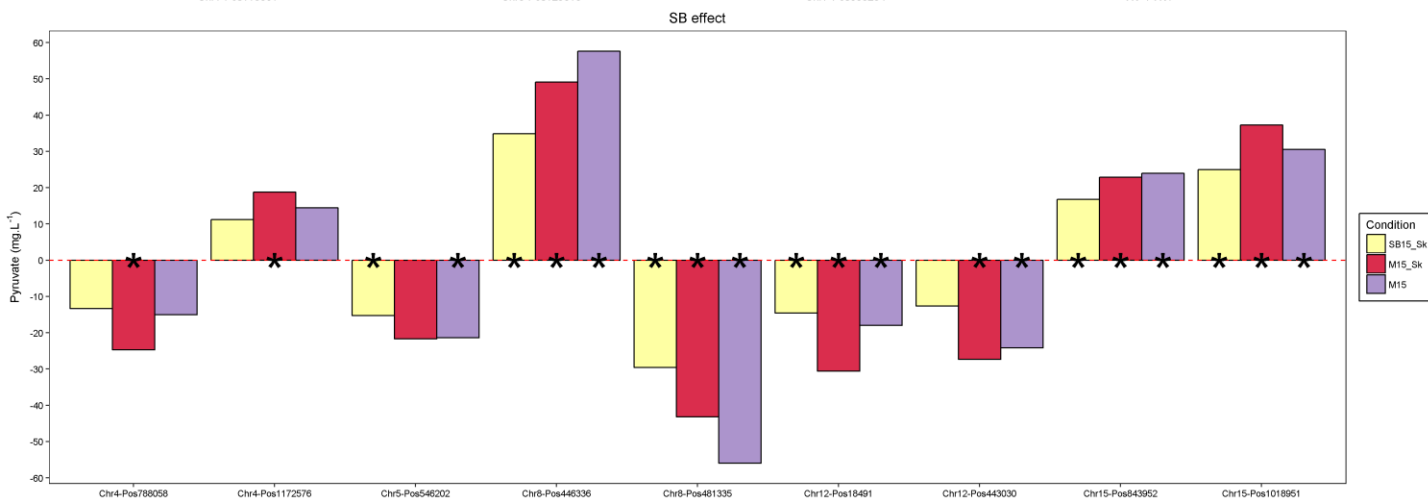

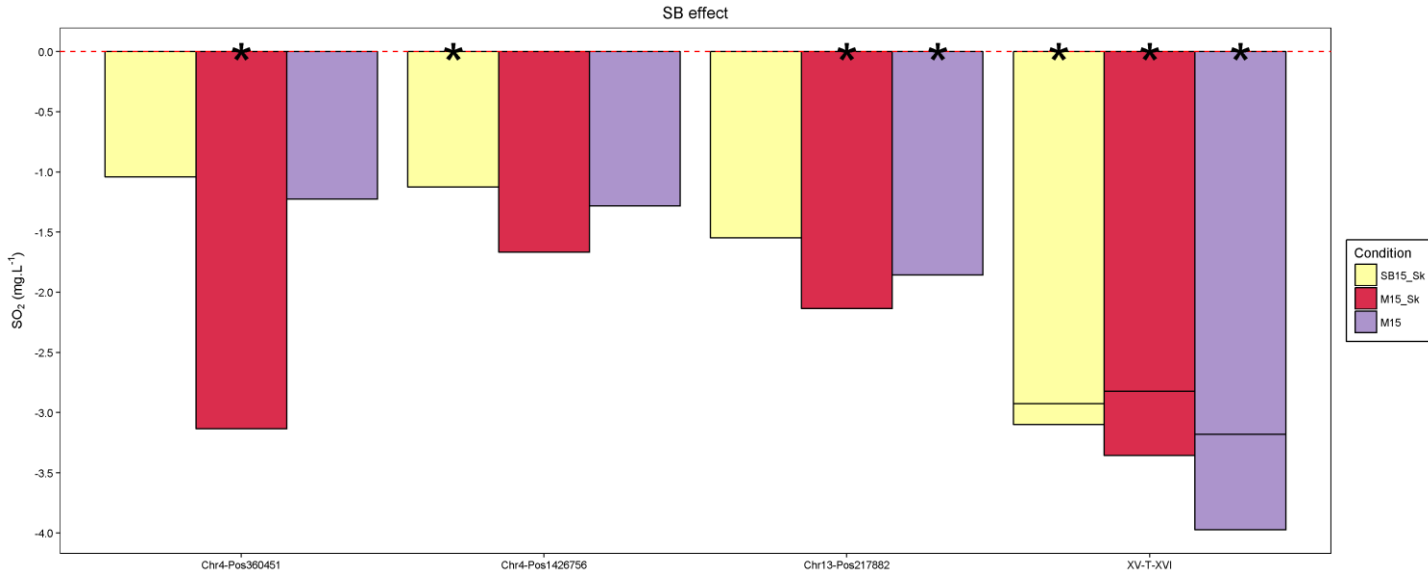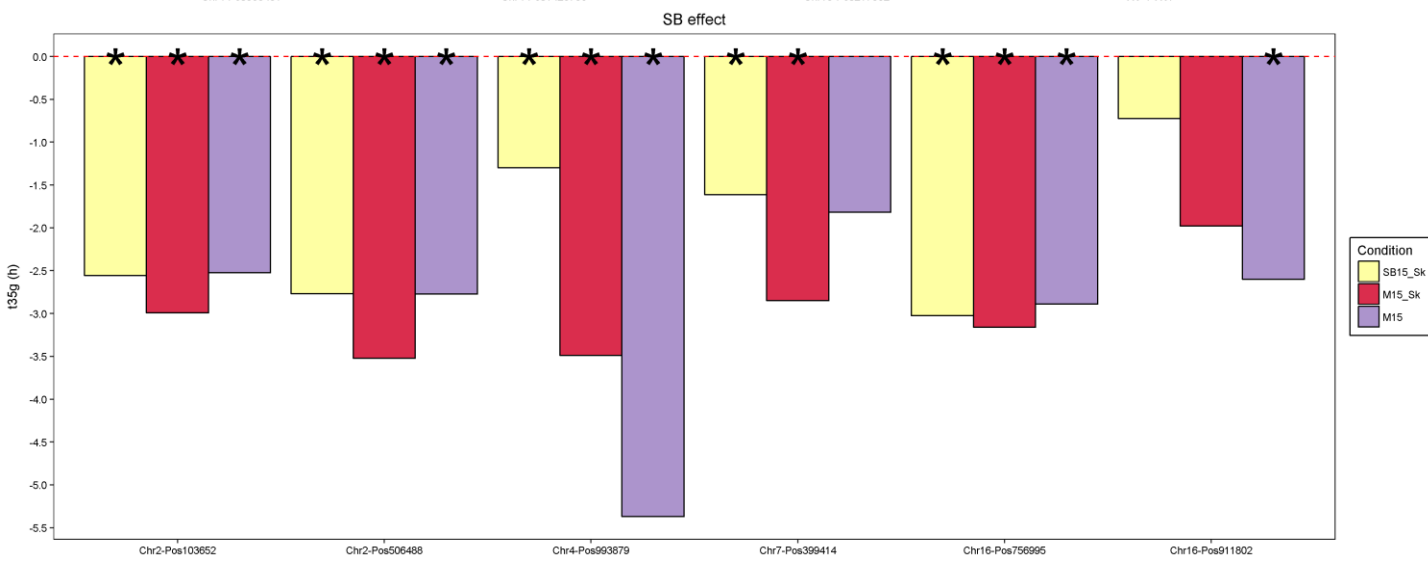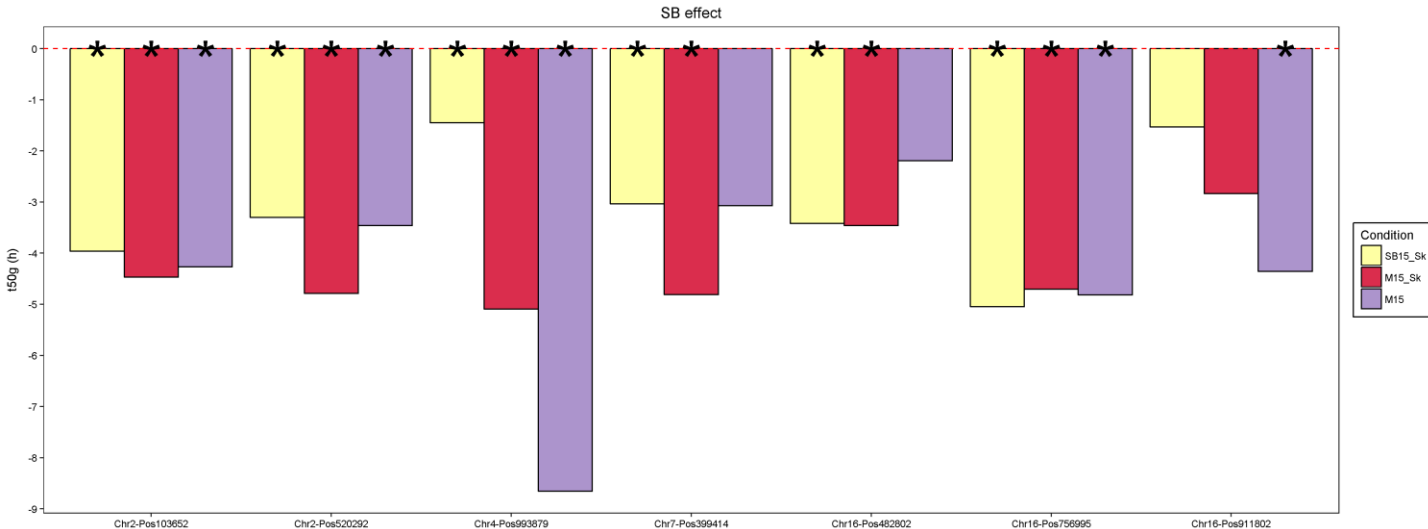

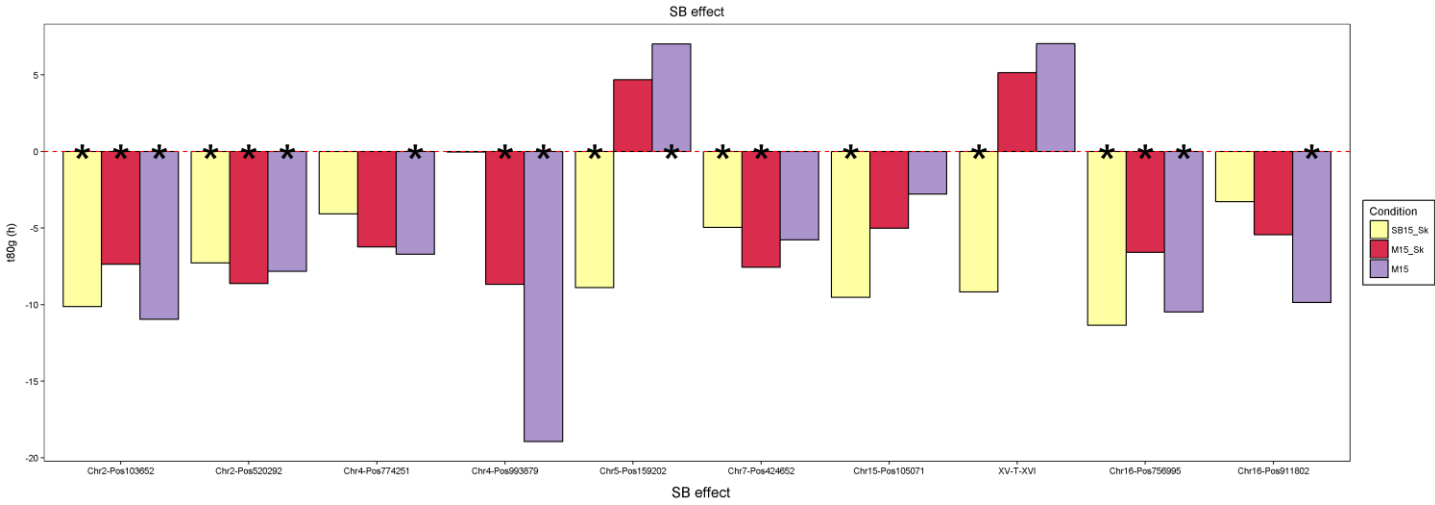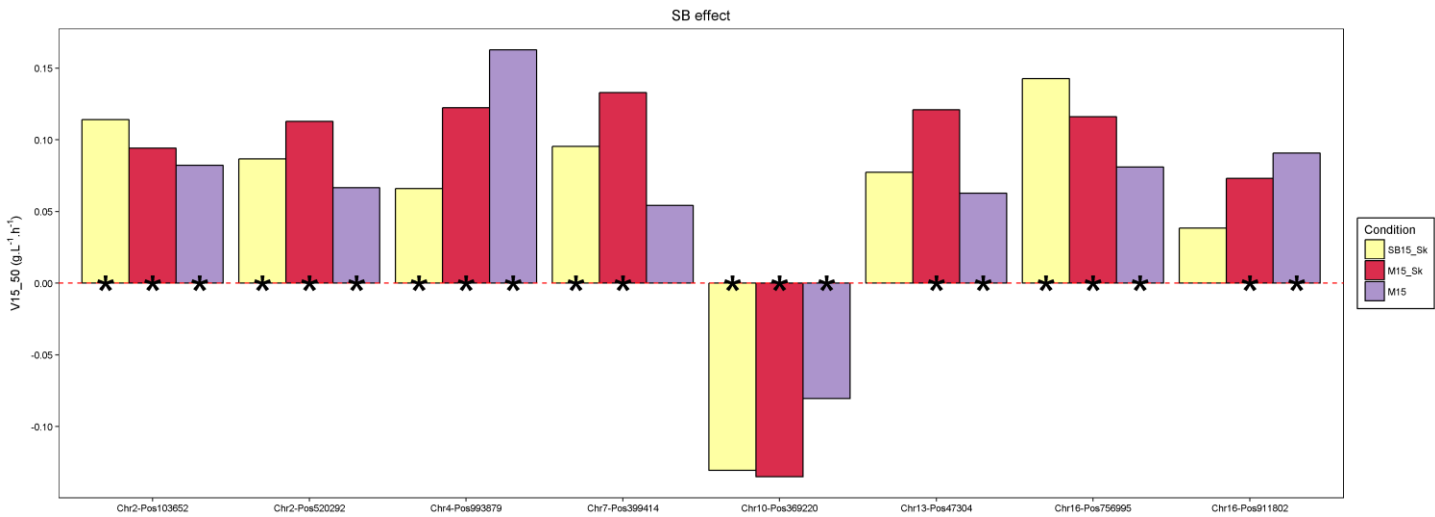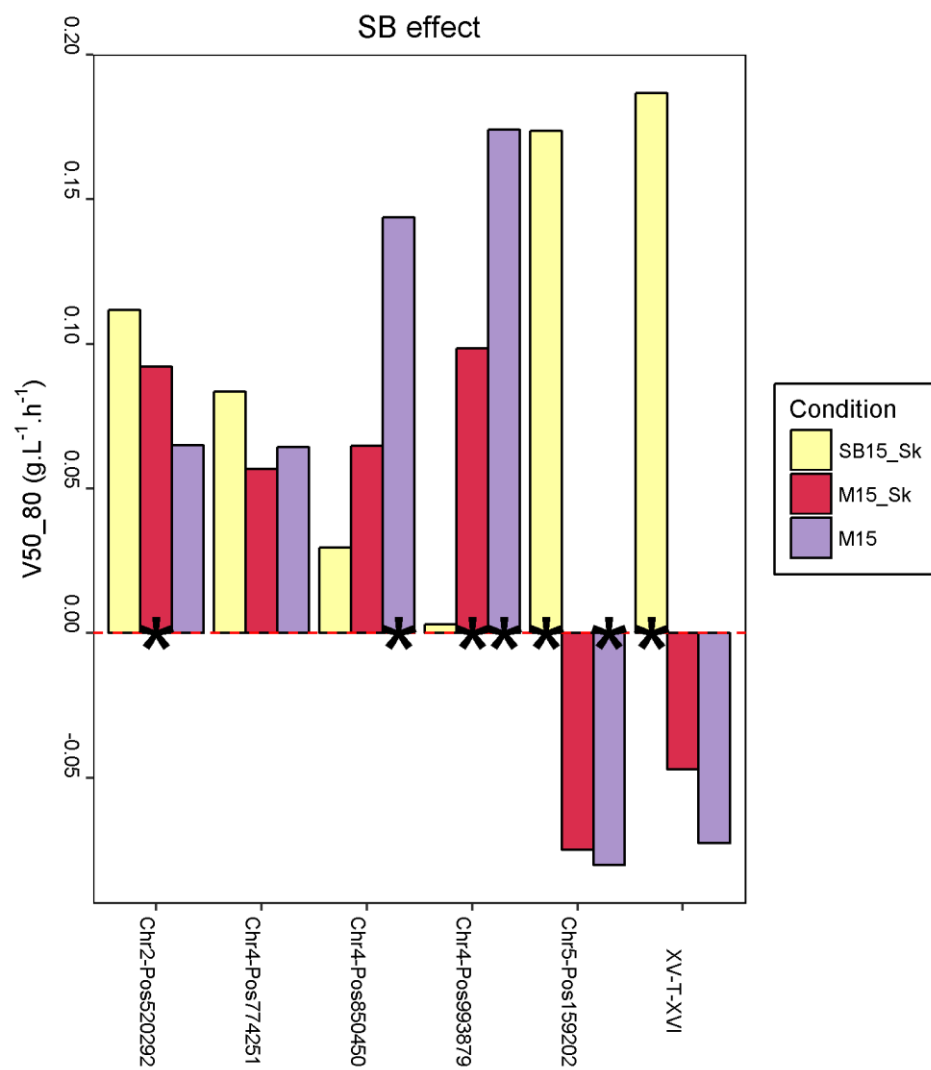

Supplement: Supplementary file 14 — Figure S7. Impact of QTL XV_M2xF15_26284 on V50_80. Panel A. The norm of reaction of the segregants that inherited from M2 or F15 for QTL XV_M2xF15_26284. Dashed lines show the reaction norm of each segregant according to their allele inheritance. Full lines show average value of the all the segregant according to marker inheritance. Panel B. Enrichment of strains having inherited the F15 allele in cluster 2. Norm of reaction of each individual is shown in dotted line and faceted according to their cluster. M2xF15 progeny clones are colored according to their allele inheritance at XV_M2xF15_26284 (red for F15 and blue for M2). A grey solid line shown the average norm of reaction of each cluster. Number of M2xF15 progeny clones within each cluster is indicated by n=; the first number indicate the number of strains from that inherit from M2 and the second one from strain that inherit from F15. A star means a disequilibrium in the theoretical homogeneous distribution 0.5 / 0.5 chis square test, α = 0.05). Panel C. Coverage difference between M2 and F15 strains within genomic region of QTL XV_M2xF15_26284. (PDF 154 kb) [file 12864_2018_5145_MOESM14_ESM.pdf]
