# Supplementary material for: Dissection of the molecular bases of genotype x environment interactions: a study of phenotypic plasticity of Saccharomyces cerevisiae in grape juices
Source: BMC Genomics. 2018 Nov 9;19:772. doi: 10.1186/s12864-018-5145-4 (PMC6225642; doi:10.1186/s12864-018-5145-4)
Supplement: Supplementary file 6 — Figure S2. Clustering of Norm of reaction for each trait. Norm of reaction of each individual is shown in dotted line and are colored and faceted according their cluster. Solid line shown the average norm of reaction of each cluster. Number of strains within each cluster is indicated by n. (PDF 2768 kb) [file 12864_2018_5145_MOESM6_ESM.pdf]

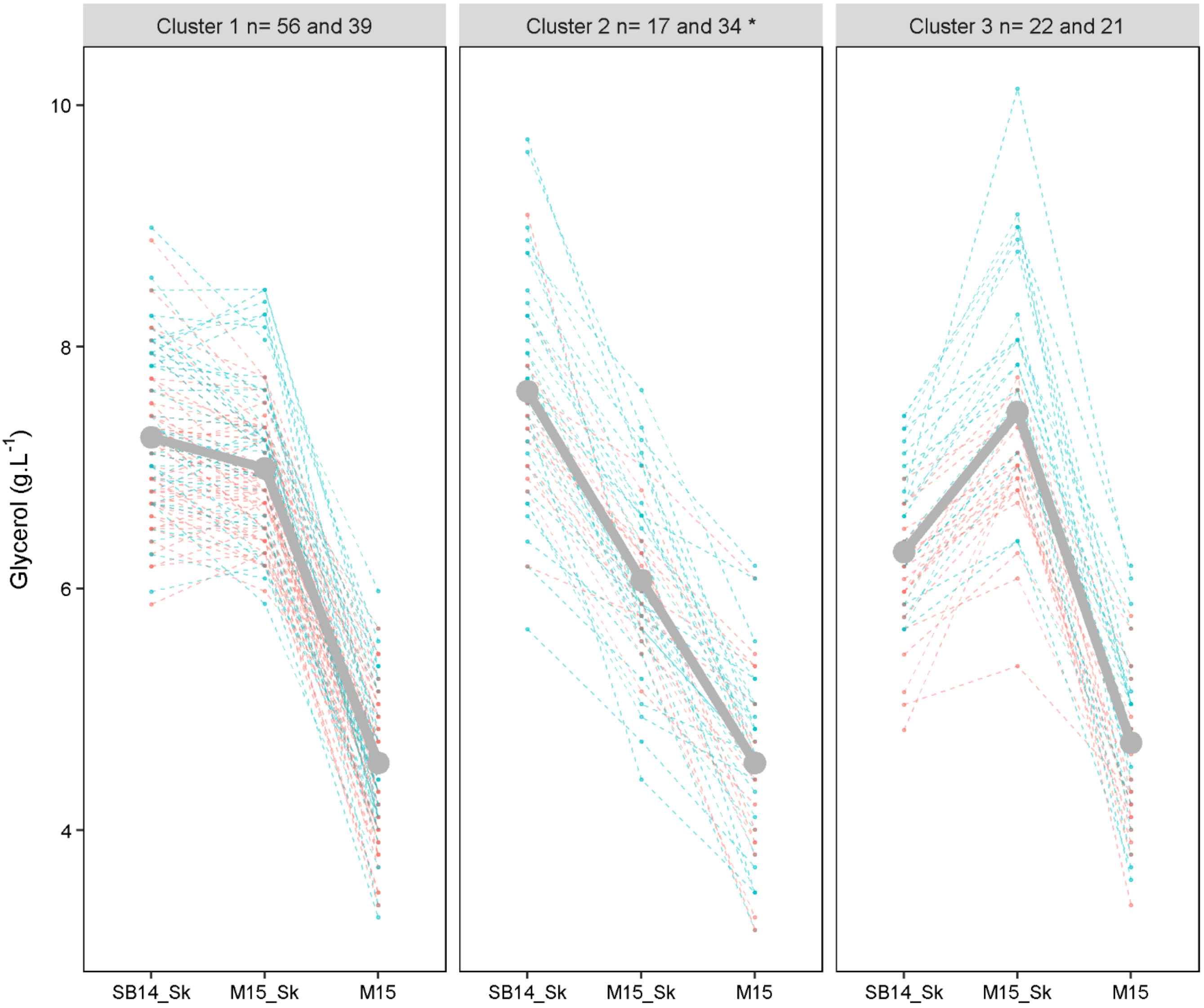

Cluster 1 n= 56 and 39
Cluster 2 n= 17 and 34 *
Cluster 3 n= 22 and 21
Glycerol (g.L-1)
10
8
6
4
SB14_Sk
M15_Sk
M15

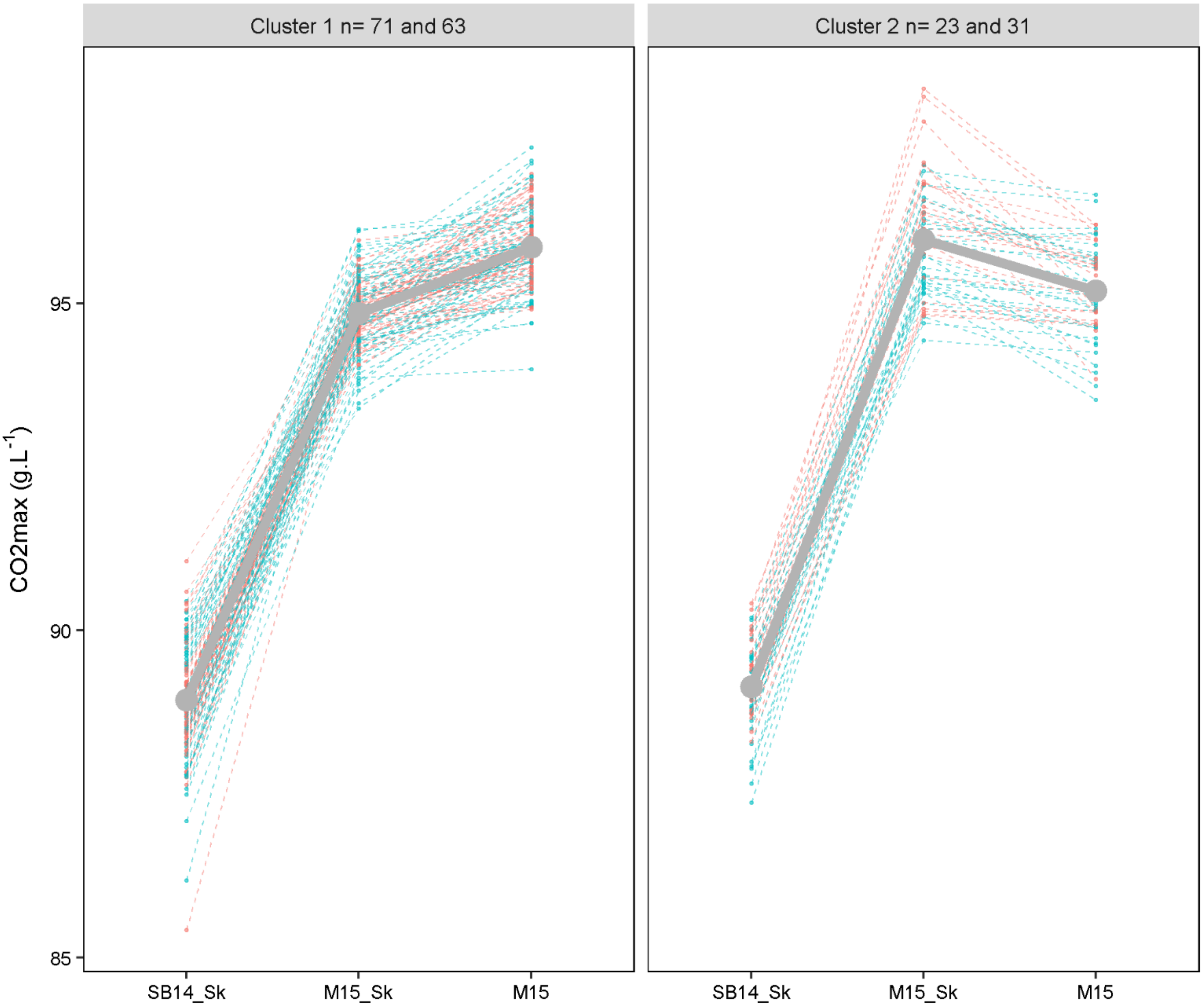
Cluster 1 n= 71 and 63
Cluster 2 n= 23 and 31
CO2max (g.L-1)
95
90
85
SB14_Sk
M15_Sk
M15

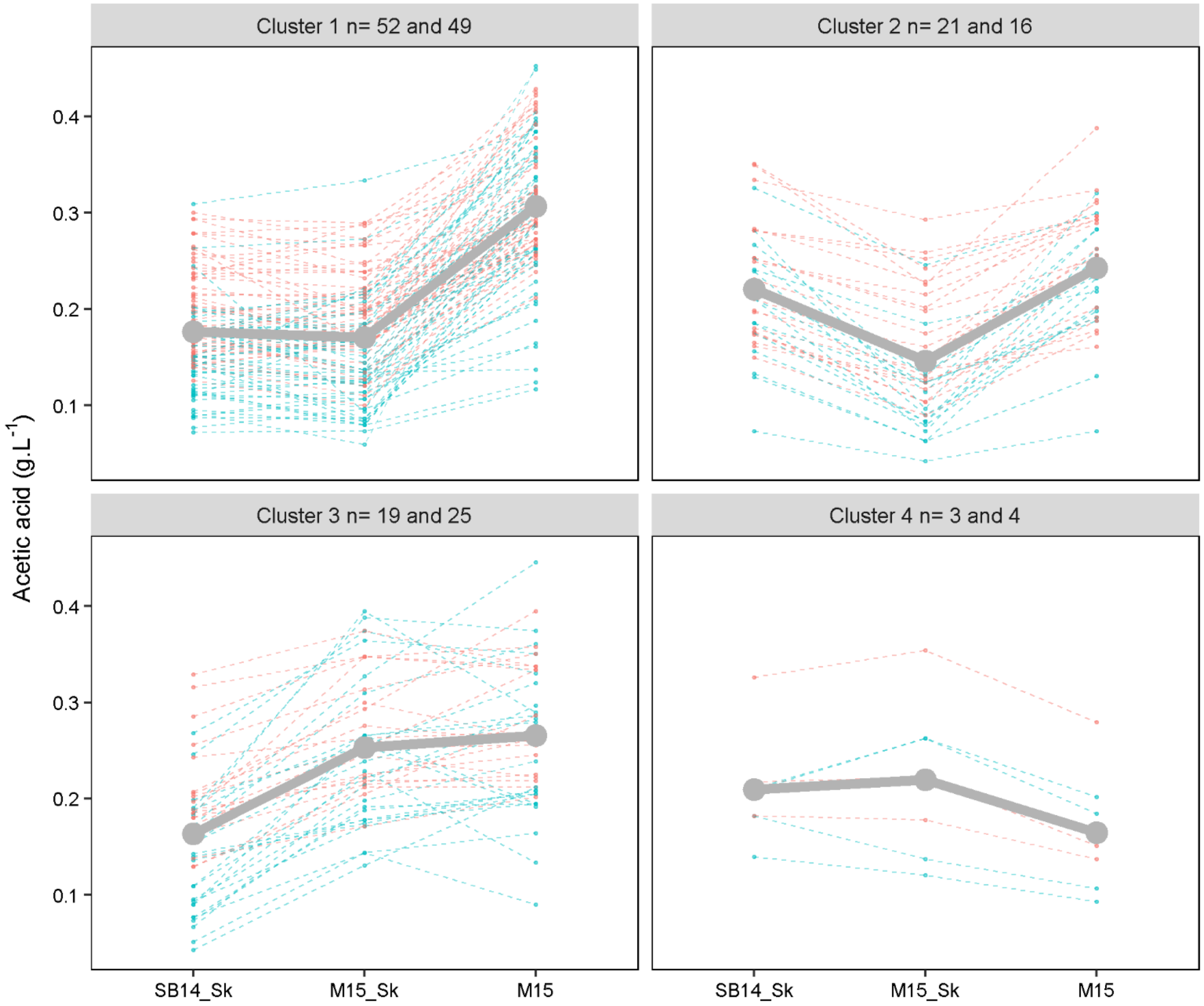
Cluster 1 n= 52 and 49
Cluster 2 n= 21 and 16
Cluster 3 n= 19 and 25
Cluster 4 n= 3 and 4
Acetic acid (g.L-1)
0.1
0.2
0.3
0.4
SB14_Sk
M15_Sk
M15

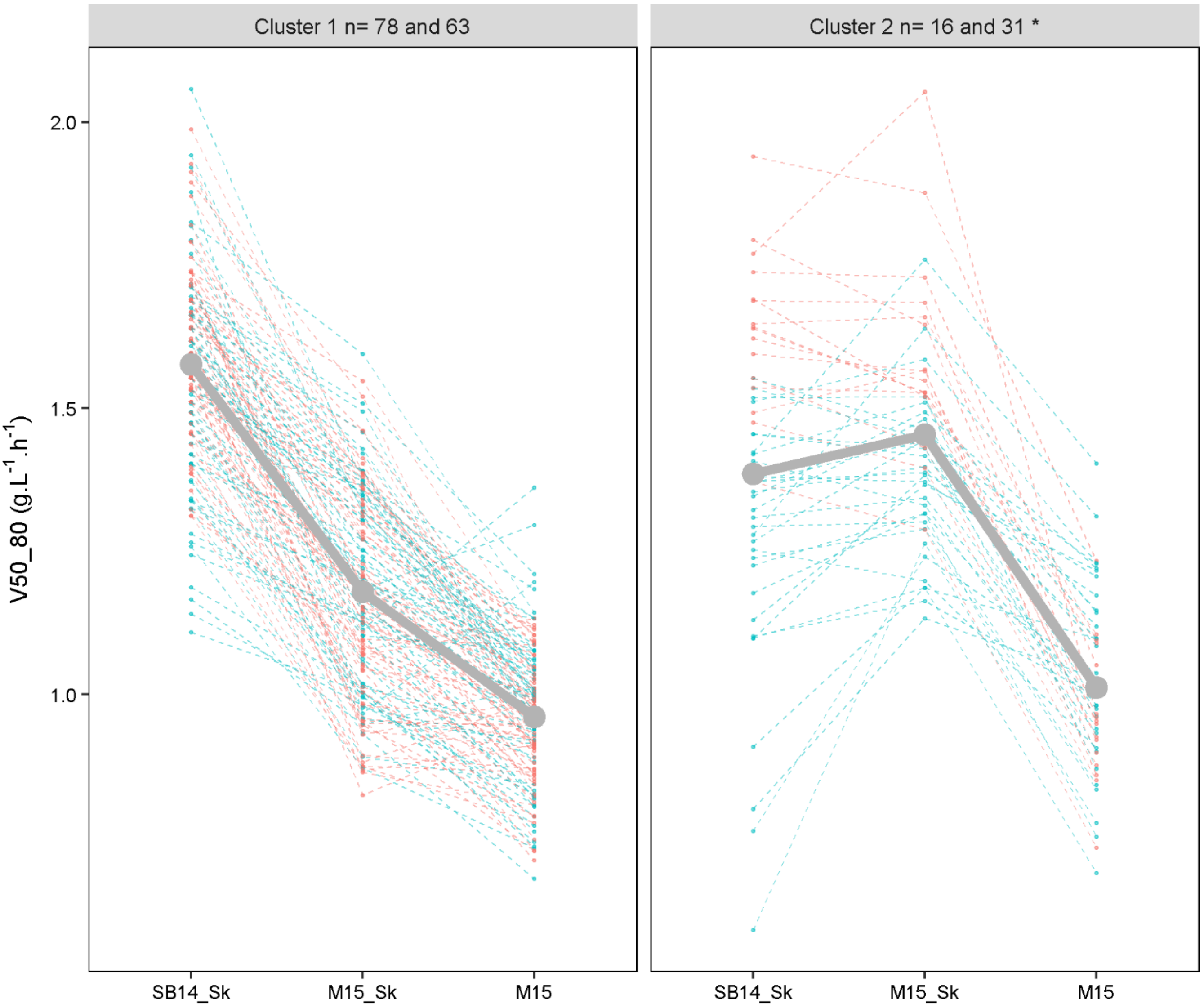

Cluster 1 n= 78 and 63
Cluster 2 n= 16 and 31 *
V50_80 (g.L-1.h-1)
2.0
1.5
1.0
SB14_Sk
M15_Sk
M15
SB14_Sk
M15_Sk
M15

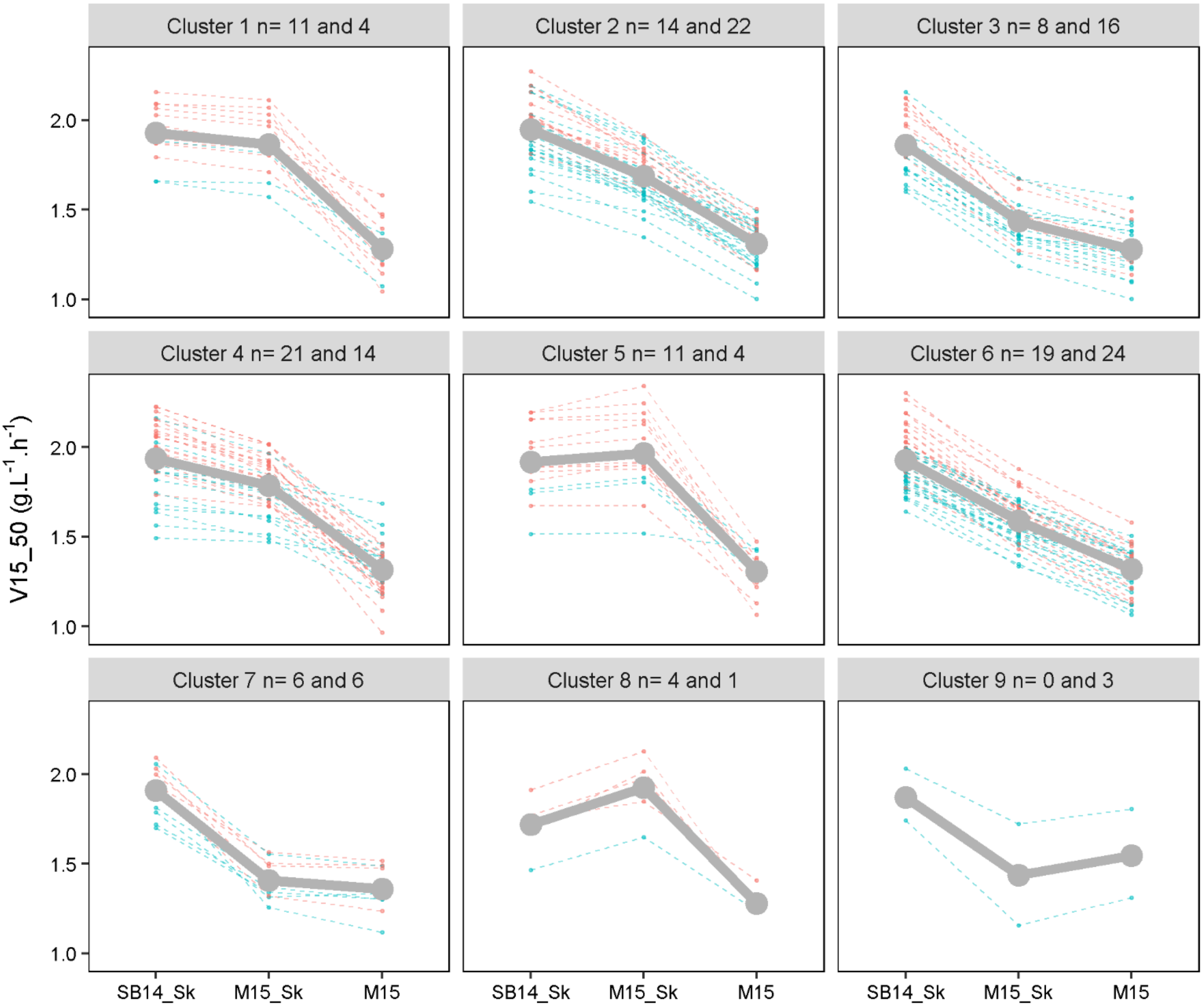
Cluster 1 n= 11 and 4
Cluster 2 n= 14 and 22
Cluster 3 n= 8 and 16
Cluster 4 n= 21 and 14
Cluster 5 n= 11 and 4
Cluster 6 n= 19 and 24
Cluster 7 n= 6 and 6
Cluster 8 n= 4 and 1
Cluster 9 n= 0 and 3
V15_50 (g.L-1.h-1)
2.0
1.5
1.0
SB14_Sk
M15_Sk
M15

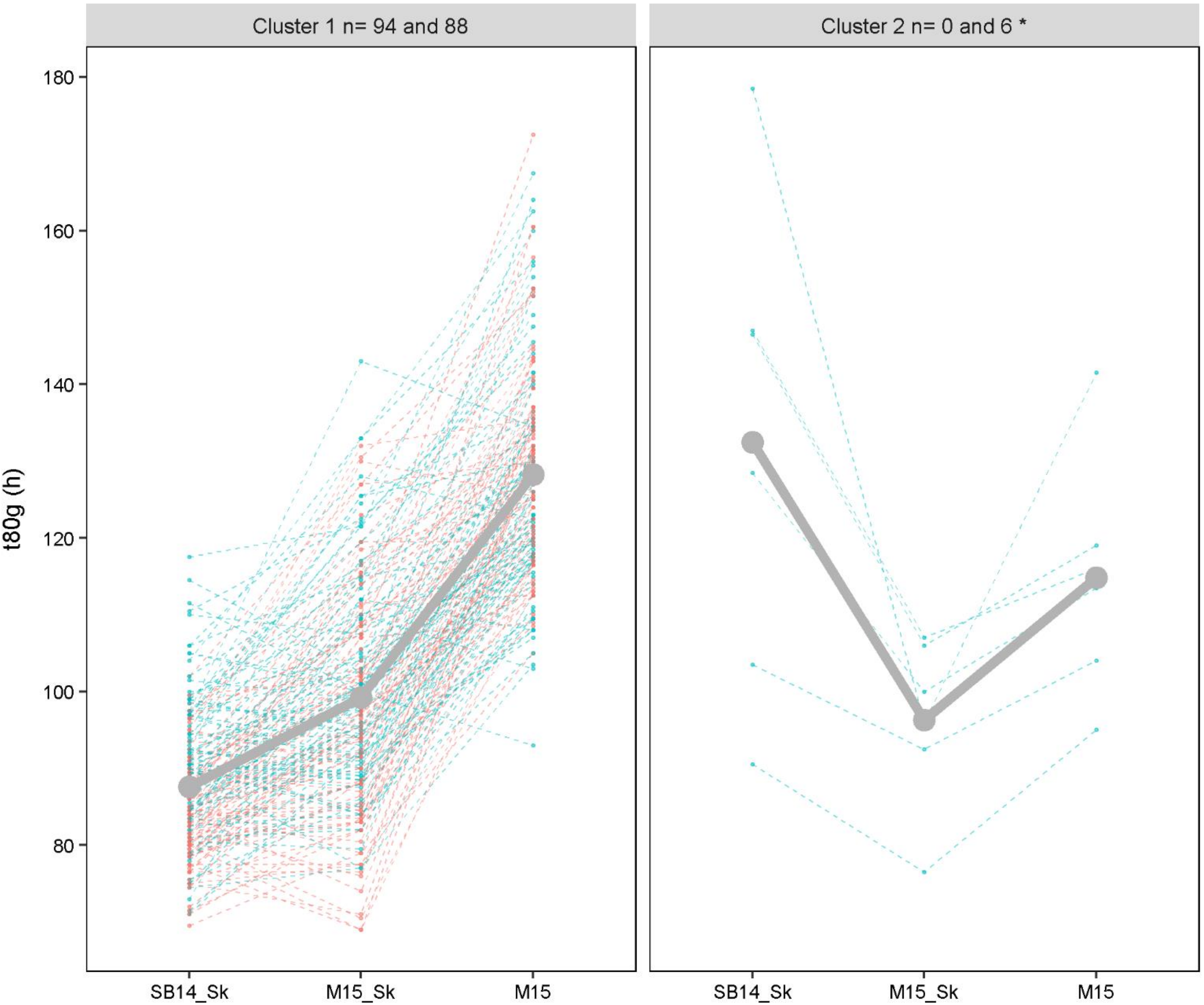

Cluster 1 n= 94 and 88
Cluster 2 n= 0 and 6 *
t80g (h)
180
160
140
120
100
80
SB14_Sk
M15_Sk
M15
SB14_Sk
M15_Sk
M15

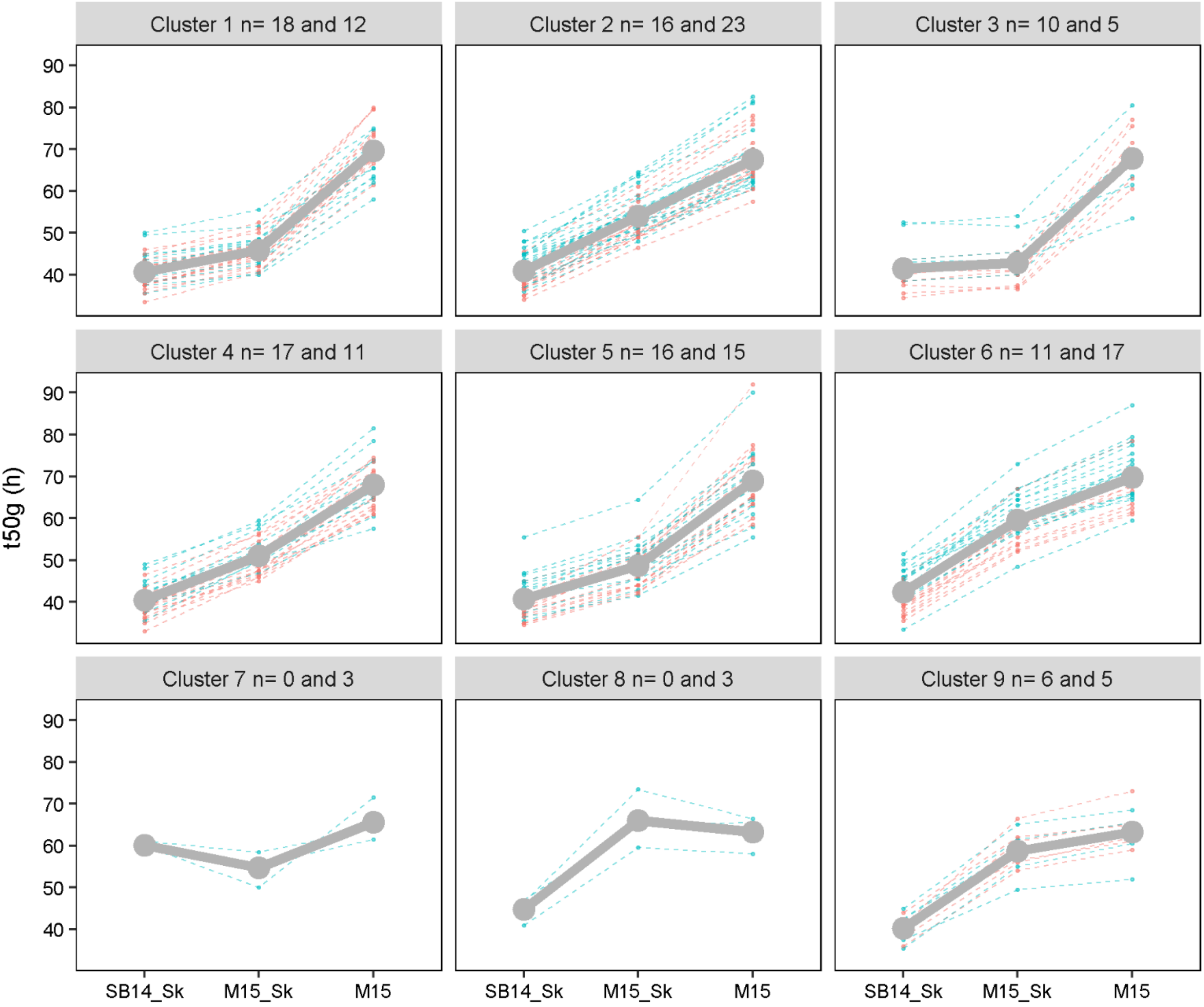
Cluster 1 n= 18 and 12
Cluster 2 n= 16 and 23
Cluster 3 n= 10 and 5
Cluster 4 n= 17 and 11
Cluster 5 n= 16 and 15
Cluster 6 n= 11 and 17
Cluster 7 n= 0 and 3
Cluster 8 n= 0 and 3
Cluster 9 n= 6 and 5
t50g (h)
90
80
70
60
50
40
SB14_Sk
M15_Sk
M15

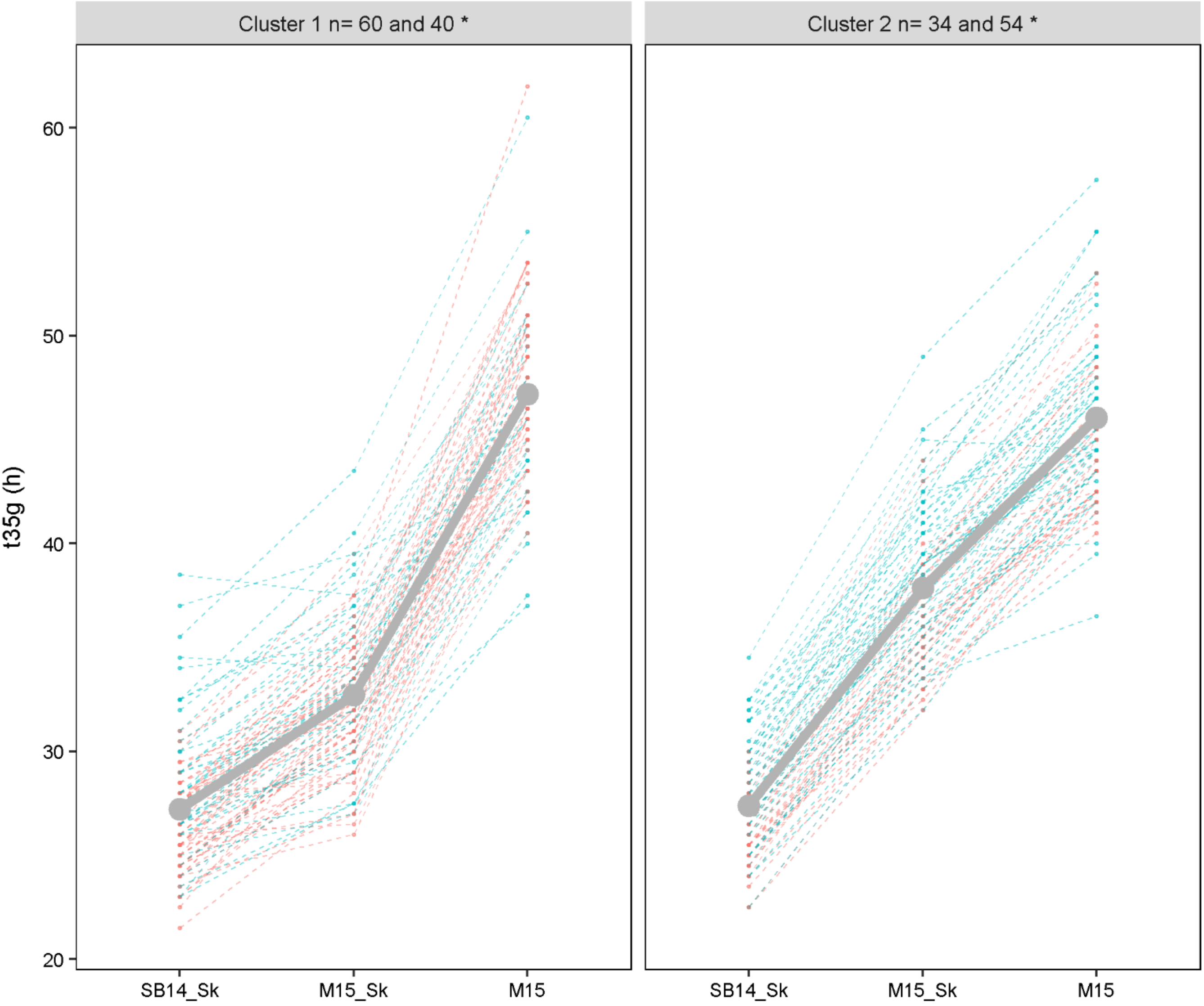

Cluster 1 n= 60 and 40 *
Cluster 2 n= 34 and 54 *
t35g (h)
60
50
40
30
20
SB14_Sk
M15_Sk
M15
SB14_Sk
M15_Sk
M15

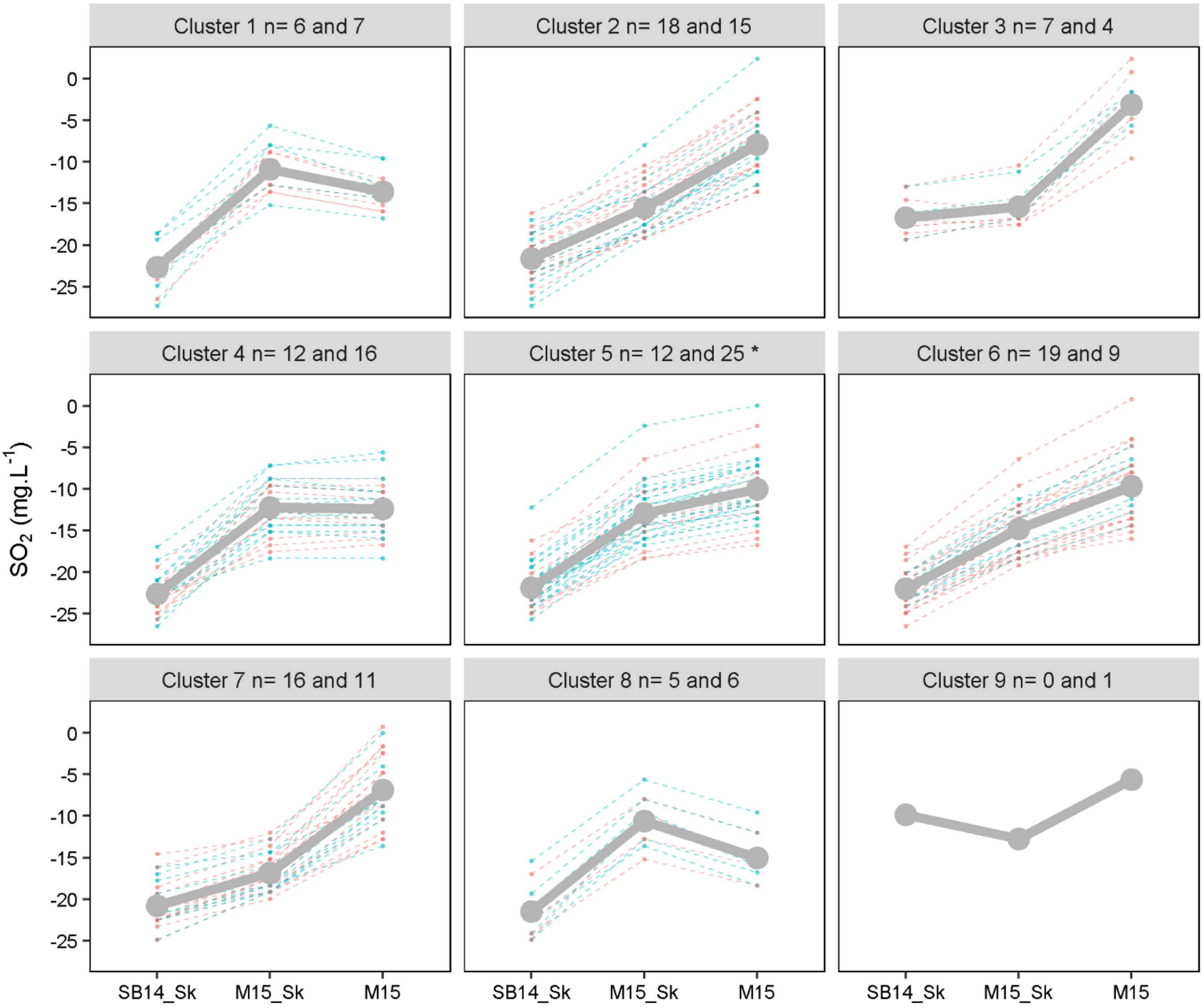

Cluster 1 n= 6 and 7
Cluster 2 n= 18 and 15
Cluster 3 n= 7 and 4
Cluster 4 n= 12 and 16
Cluster 5 n= 12 and 25 *
Cluster 6 n= 19 and 9
Cluster 7 n= 16 and 11
Cluster 8 n= 5 and 6
Cluster 9 n= 0 and 1
SO_2 (mg.L^{-1})
0
-5
-10
-15
-20
-25
SB14_Sk
M15_Sk
M15

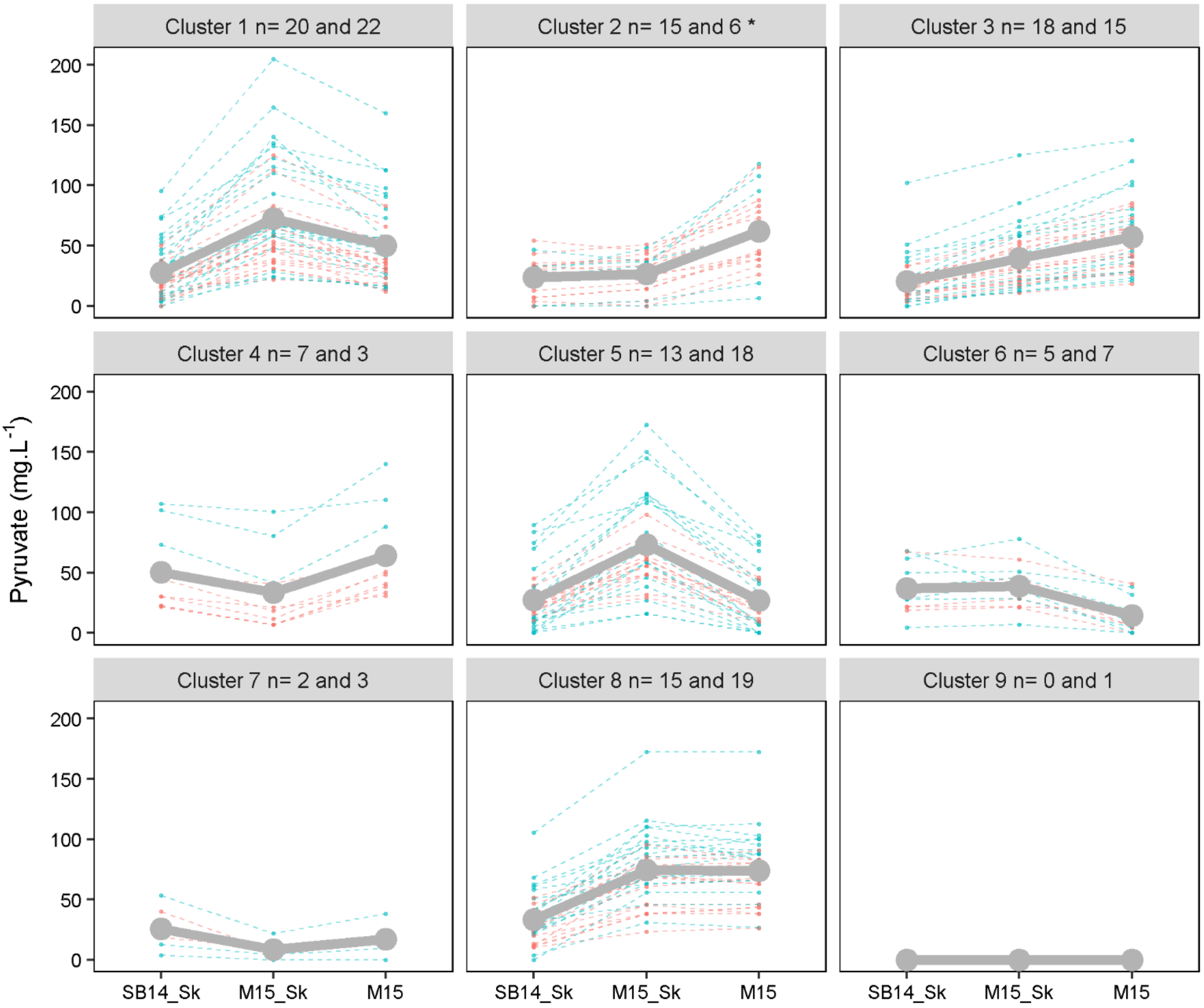
Cluster 1 n= 20 and 22
Cluster 2 n= 15 and 6 *
Cluster 3 n= 18 and 15
Cluster 4 n= 7 and 3
Cluster 5 n= 13 and 18
Cluster 6 n= 5 and 7
Cluster 7 n= 2 and 3
Cluster 8 n= 15 and 19
Cluster 9 n= 0 and 1
Pyruvate (mg.L-1)
0
50
100
150
200
SB14_Sk
M15_Sk
M15

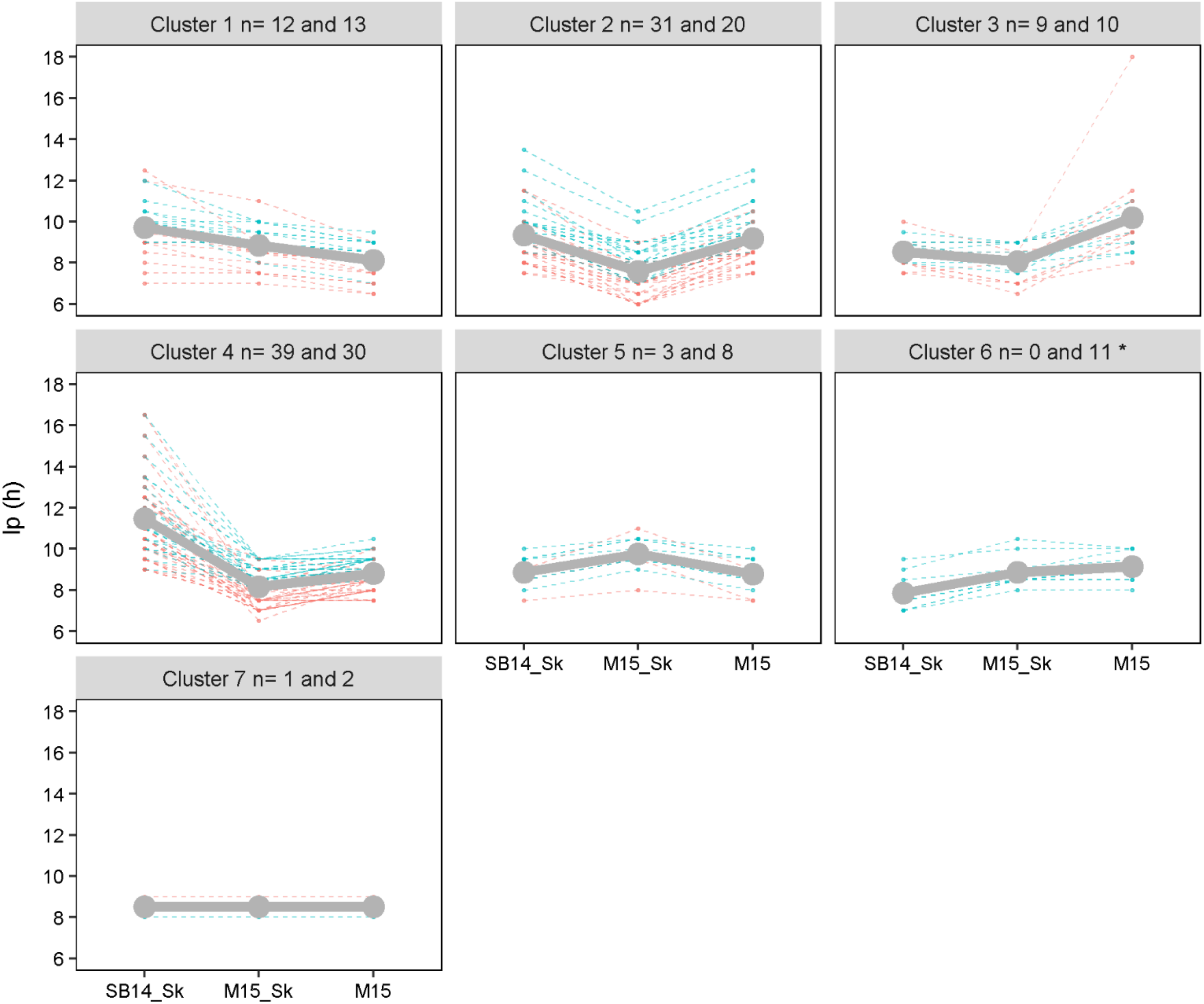
Cluster 1 n= 12 and 13
Cluster 2 n= 31 and 20
Cluster 3 n= 9 and 10
Cluster 4 n= 39 and 30
Cluster 5 n= 3 and 8
Cluster 6 n= 0 and 11 *
Cluster 7 n= 1 and 2
Ip (h)
6
8
10
12
14
16
18
SB14_Sk
M15_Sk
M15
